# Structural, electronic and kinetic properties of the phase-change material Ge_2_Sb_2_Te_5_ in the liquid state

**DOI:** 10.1038/srep27434

**Published:** 2016-06-08

**Authors:** Mathias Schumacher, Hans Weber, Pál Jóvári, Yoshimi Tsuchiya, Tristan G. A. Youngs, Ivan Kaban, Riccardo Mazzarello

**Affiliations:** 1 Institute for Theoretical Solid State Physics, RWTH Aachen University, 52056 Aachen, Germany; 2 IFW Dresden, Institute for Complex Materials, PO Box 270116, 01171 Dresden, Germany; 3 TU Dresden, Institut für Strukturphysik, 01062 Dresden, Germany; 4 Wigner Research Centre for Physics, Institute for Solid State Physics and Optics, PO Box 49, 1525 Budapest, Hungary; 5 Department of Physics, Faculty of Science, Niigata University, Ikarashi 2-8050, Niigata 950-21, Japan; 6 ISIS Facility, STFC Rutherford Appleton Laboratory, Harwell Oxford, Didcot, UK; 7 JARA-FIT and JARA-HPC, RWTH Aachen University, 52056 Aachen, Germany

## Abstract

Phase-change materials exhibit fast and reversible transitions between an amorphous and a crystalline state at high temperature. The two states display resistivity contrast, which is exploited in phase-change memory devices. The technologically most important family of phase-change materials consists of Ge-Sb-Te alloys. In this work, we investigate the structural, electronic and kinetic properties of liquid Ge_2_Sb_2_Te_5_ as a function of temperature by a combined experimental and computational approach. Understanding the properties of this phase is important to clarify the amorphization and crystallization processes. We show that the structural properties of the models obtained from *ab initio* and reverse Monte Carlo simulations are in good agreement with neutron and X-ray diffraction experiments. We extract the kinetic coefficients from the molecular dynamics trajectories and determine the activation energy for viscosity. The obtained value is shown to be fully compatible with our viscosity measurements.

Due to continuously increasing demand on high-density and fast data-storage devices, the interest in phase-change materials (PCMs) and their applications in non-volatile data storage media is extremely high^1^,^2^,^3^. The first work on PCMs dates back to 1968, when Ovshinsky reported on a sharp transition from high resistivity to low resistivity in amorphous Te_48_As_30_Si_12_Ge_10_ induced by an electric field (electric switching)^4^. However, another property of PCMs – the large contrast in the optical reflectivity between the amorphous and crystalline state – led to practical applications in the data storage media, namely in rewritable optical discs such as CDs, DVDs and Blu-ray discs. Many such devices are based on the Ge-Sb-Te (GST) PCM alloys from the pseudo-binary line between GeTe and Sb_2_Te_3_ compounds, discovered by Yamada *et al.*^5^.

Recently, the development of the phase-change random access memory (PCRAM) has sparked renewed interest in the electric switching phenomenon. PCRAMs exploit the electronic contrast between the amorphous and crystalline state. It is plausible that these non-volatile devices will be able to outperform and replace the Flash memory, whose miniaturization will soon come to an end due to physical limits. At present, GST alloys are among the most promising PCMs for high-speed, high-reliability PCRAMs. In spite of intensive studies, however, a complete understanding of the switching phenomena and the property contrast at the atomic level, which is required for the development of new, better performing data-storage media, is still lacking. Furthermore, the elucidation of the relations between the chemical composition, atomic structure, bonding mechanism and resulting phase-change properties remains a challenge for physicists and material scientists.

Apart from the technological importance of PCMs, the peculiar combination of physical properties of the crystalline, amorphous and liquid state is of great scientific interest too^1^,^2^,^3^. During the last years, significant progress has been made in the theoretical and experimental investigation of both crystalline^6^,^7^,^8^ and amorphous^9^,^10^,^11^,^12^,^13^,^14^,^15^,^16^,^17^,^18^ GST alloys, while our knowledge of the structural and dynamical properties of the liquid state is more limited^19^,^20^,^21^,^22^,^23^,^24^,^25^,^26^,^27^,^28^. In particular, there are still open questions about the crystalline-to-amorphous (amorphization) and amorphous-to-crystalline (devitrification) phase transitions, which can be clarified only by a detailed study of the liquid state. In PCM-based devices, the amorphization process occurs due to local melting (by a laser or current pulse) and subsequent quenching from the melt. It has been recently understood^29^,^30^,^31^,^32^ that the high fragility of the supercooled liquid state of PCMs plays a key role in the devitrification process. Fragility describes the deviation of the temperature dependence of the viscosity *η* from the Arrhenius behaviour. The high fragility of PCMs is responsible for two essential properties of PCMs, namely the rapid crystallization at high temperature and the high stability of the amorphous phase at room temperature. Hence, knowledge of the atomic structure and the dynamics of Ge-Sb-Te alloys in the liquid and supercooled liquid state is indispensable for a complete comprehension of the phase-change processes.

*Ab initio* molecular dynamics (AIMD) simulations based on density functional theory (DFT) have been shown to yield models of PCMs which, overall, are in good agreement with experimental data. For standard generalized-gradient-approximation (GGA) functionals, the main discrepancy is the fact that the bond distances in liquid and amorphous PCMs are typically overestimated by 1–3%. A number of studies, including some of our own works^12^,^13^,^33^,^34^,^35^,^36^,^37^, have shown that the complementary information provided by different experimental techniques such as X-ray diffraction (XRD), neutron diffraction (ND) and extended X-ray absorption fine structure (EXAFS) can be effectively combined and interpreted in the frame of the reverse Monte-Carlo (RMC) simulation method^38^. In particular, in refs 12,13, the RMC method was used to analyse XRD, ND and EXAFS data for amorphous Ge_1_Sb_2_Te_4_ and Ge_2_Sb_2_Te_5_ alloys. These studies have provided very detailed information on the local atomic structure of these amorphous systems.

In this work, we investigate the atomic ordering in the liquid phase of Ge_2_Sb_2_Te_5_, as well its electronic and kinetic properties, by combining AIMD simulations with experiments and RMC simulations. We use AIMD and a van der Waals density functional which includes non-local correlations^39^ (denoted as vdW-DF2) to generate long trajectories at different temperatures, from which we extract the structural properties (including structure factors, pair and three-body correlation functions, and ring statistics), the electronic structure and the viscosity coefficients of the liquid state. These quantities are compared with experimental XRD data, neutron diffraction with Ge isotopic substitution data, and viscosity measurements. The AIMD structural models are also taken as input for refined RMC simulations. We show that the agreement between simulations and experiments is quite good. In particular, the mismatch between experimental and theoretical atomic distances is partially cured by the use of the vdW-DF2 functional and the temperature dependence of the activation energy for the viscosity matches the experimental value nicely. We also discuss the discrepancies in the magnitude of the viscosity in relation to the approximations made in our simulations.

## Results

### Structural and electronic properties

**Figure 1:**
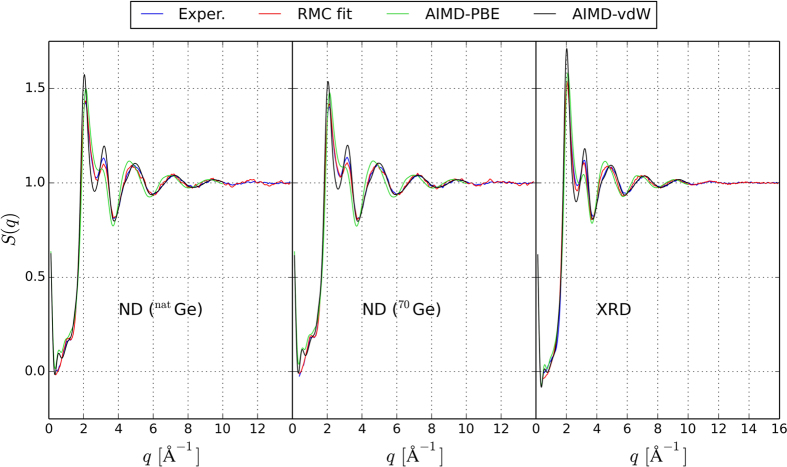
X-ray and neutron diffraction total structure factors *S*(*q*) for liquid Ge_2_Sb_2_Te_5_ measured at 923 K (blue curves) compared to the structure factors obtained from vdW-DF2 and GGA AIMD simulations at 925–926 K (black and green curves respectively) and RMC simulations (red curves). The AIMD *S*(*q*) are computed from the partial structure factors *S*_*ij*_(*q*) weighted by the X-ray or neutron atomic form factors. The partial factors *S*_*ij*_(*q*) are calculated by Fourier transforming the partial pair correlation functions (shown in Fig. 2).

**Figure 2:**
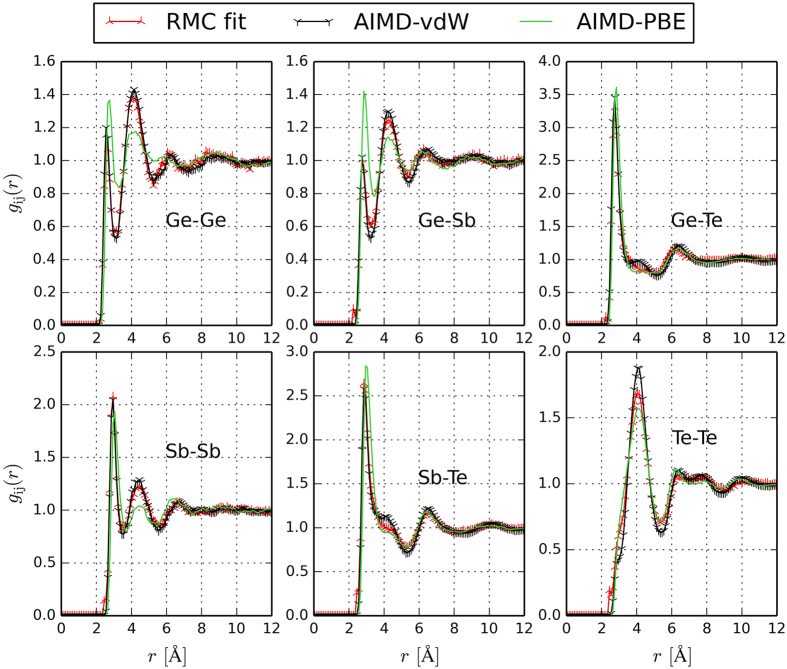
RMC (*T* = 923 K), vdW-DF2 AIMD (*T* = 925 K) and GGA AIMD (*T* = 926 K) partial pair distribution functions *g*_*ij*_(*r*) for liquid Ge_2_Sb_2_Te_5_.

The experimental neutron and X-ray total structure factors *S*(*q*) for liquid Ge_2_Sb_2_Te_5_ measured at 923 K (which is close to the melting point, *T* = 900 K) are compared with AIMD and RMC structure factors in Fig. 1. As the AIMD models are more informative than the RMC configurations, the former are discussed in detail in the following. The AIMD models contain 540 atoms and their density is set to 0.030 Å^−3^, which is an average over the values we determined experimentally by a high energy γ-ray attenuation method in the temperature range considered (see Table 1). To generate the models, we employ the following procedure: we start from a random configuration and equilibrate the system at *T* = 3000 K for 30 ps. Then, we quench the system to the target temperature (925 K) and equilibrate for 30 ps. After equilibration, we compute the total partial distribution function *g*(*r*), as well as the six partial pair distribution functions *g*_*ij*_(*r*) (where *i* and *j* indicate the atomic species) by averaging over 20 ps. The AIMD partial *g*_*ij*_(*r*)’s are plotted in Fig. 2, together with the RMC fits. For the sake of comparison, we also perform AIMD simulations using a standard GGA functional and include the data in Figs 1 and 2. Notice that the three experimental data sets are not sufficient to separate the 6 partial correlation functions of this 3 component system. We calculate the neutron and X-ray structure factor *S*(*q*) by combining the partial structure factors *S*_*ij*_(*q*) (obtained from the Fourier transforms of the *g*_*ij*_(*r*) functions) with the X-ray and neutron diffraction weights. Similar to the experimental structure factors, the AIMD total *S*(*q*) curves exhibit a pronounced first peak and a smaller second peak. This is indicative of octahedral order, as discussed in ref. 21, where ND measurements were performed on several GST liquid alloys and compared with other octahedral liquids, as well as with selected tetrahedral liquids. For this purpose, the order parameter *S* ≡ *S*(*q*_*2*_)*/S*(*q*_*1*_) was introduced, where *q*_*1*_ and *q*_*2*_ denote the position of the first and second peak^21^. Our AIMD models yield *S* = 0.69. The intensity of the first and second peak of the theoretical *S*(*q*) is overestimated as compared to experiments. We also observe a small shift of the peaks to lower values of *q*. As far as the third and subsequent peaks are concerned, the agreement is excellent. Overall, the curves are closer to experimental values than those obtained using GGA functionals (except for the height of the first peak), which lead to an underestimation of the second peak and a pronounced shift of the third peak to lower diffraction vectors. Notice that there is very good correspondence of the RMC fits with the experimental datasets.

**Figure 3:**
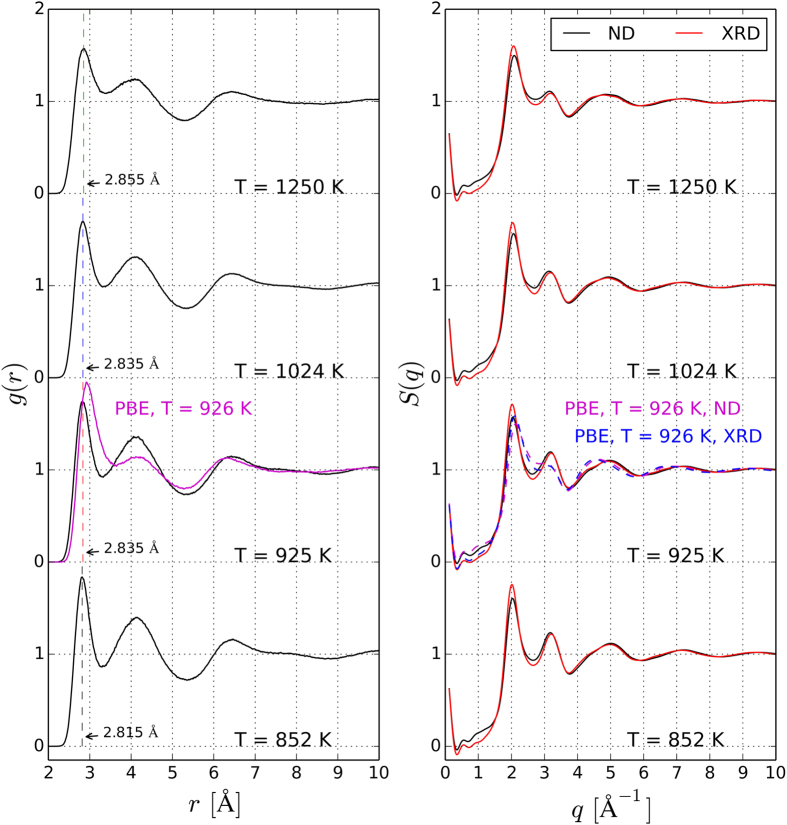
Total radial distribution functions *g*(*r*) (left panel) and total structure factors *S*(*q*) (right panel) of liquid Ge_2_Sb_2_Te_5_ obtained from AIMD simulations. Temperatures range from 852 K up to 1250 K. The corresponding partial pair correlation functions *g*_*ij*_(*r*) are shown in Fig. 2 (*T* = 923 K) and in the supplement (*T* = 852, 1024 and 1250 K). Solid red lines are drawn for the X-ray diffraction structure factors, whereas the *S*(*q*) for neutron diffraction are indicated by black lines. In the left panel, vertical dashed lines indicate the position of the first maximum of the vdW-DF2 *g*(*r*), underlining the shift to larger particle-particle distances with increasing temperature. GGA AIMD data at *T* = 926 K are also included. In ref. 27, GGA functionals with van der Waals semi-empirical corrections were employed and a first-peak position of 2.79 Å was obtained for supercooled liquid Ge_2_Sb_2_Te_5_ at *T* = 820 K.

We also perform AIMD simulations at three other temperatures, namely at *T* = 852, 1024 and 1250 K. Since the melting temperature of Ge_2_Sb_2_Te_5_ is about 900 K, the lowest temperature considered corresponds to a slightly supercooled liquid phase. The total pair distribution functions *g*(*r*) are presented in Fig. 3, together with the structure factors, whereas the partial *g*_*ij*_(*r*)’s at 852, 1024 and 1250 K are shown in the supplement, Figs S1–3. We also include the GGA curves at *T*  =  926 K. The latter are in very good agreement with those of ref. 20. Due to the use of the vdW-DF2 functional, which yields a better description of dispersive interactions, the *g*(*r*) functions exhibit more pronounced secondary peaks and deeper minima than those obtained from standard GGA calculations. In particular, the second peak at 4.055 Å and the first and second minima at 3.325 and 5.275 Å are better defined, indicating better resolved atomic connectivities and neighbour shells. Moreover, there is a shift of the first peak to smaller interatomic distances – 2.835 Å at 925K (vdW-DF2 simulations), to be compared with 2.92 Å (GGA simulations) – which partially cures the tendency of GGA functionals to yield too long first-neighbour distances for amorphous and liquid PCMs. In refs 27,28, van der Waals semi-empirical corrections^40^ to GGA functionals were shown to have similar effects on liquid GeTe_4_ and supercooled liquid Ge_2_Sb_2_Te_5_.

We evaluate the average coordination number (CN) for each atomic species by integrating the corresponding partial distribution function up to the first minimum (see Table 2). The total and partial CNs increase with temperature. We observe a significant number of Ge-Ge and Ge-Sb bonds, as evidenced by the corresponding partial CNs included in Table 2. Recently, some of us have explained the presence of Ge-Ge bonds in the liquid phase of the parent compound GeTe as due to the small heat of formation of this compound^41^. These very bonds turn out to be responsible for the presence of tetrahedral structures in rapidly quenched amorphous GeTe^41^,^42^. We expect that similar behaviour occurs for Ge_2_Sb_2_Te_5_. Te-Te bonds are also present in the liquid state but their number decreases more drastically upon quenching to the amorphous state^14^,^15^. If one compares our CNs for Ge_2_Sb_2_Te_5_ with previous results in the literature, one observes deviations from the values at *T* = 900 K provided in ref. 20. These discrepancies stem from the use of different functionals and the different cut-off distances employed. There are also some deviations between our data at *T* = 852 K and the CNs at *T* = 822 K provided in ref. 28, which indicate that the Grimme corrections give a larger fraction of Ge-Ge and Ge-Sb bonds than vdW-DF2 functionals.

**Figure 4:**
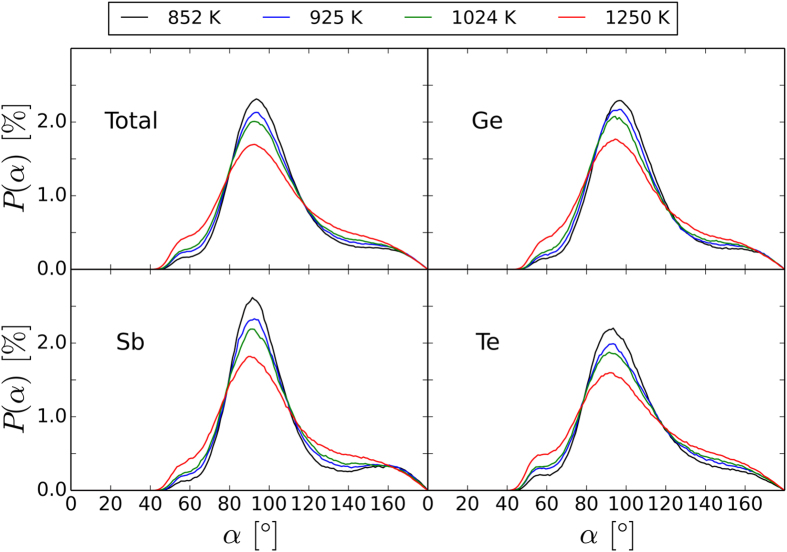
Normalized total BADs and BADs resolved over different central atoms obtained from the vdW-DF2 AIMD simulations for temperatures between 850 K and 1250 K. As temperature rises, the Ge-, Sb-, and Te-centered distributions broaden to a similar extent. Furthermore, the main peak of the Ge-resolved BADs shifts towards 90°. For an overview of the contributions to the BADs from different bond pairs, see Figs S4–7.

**Figure 5:**
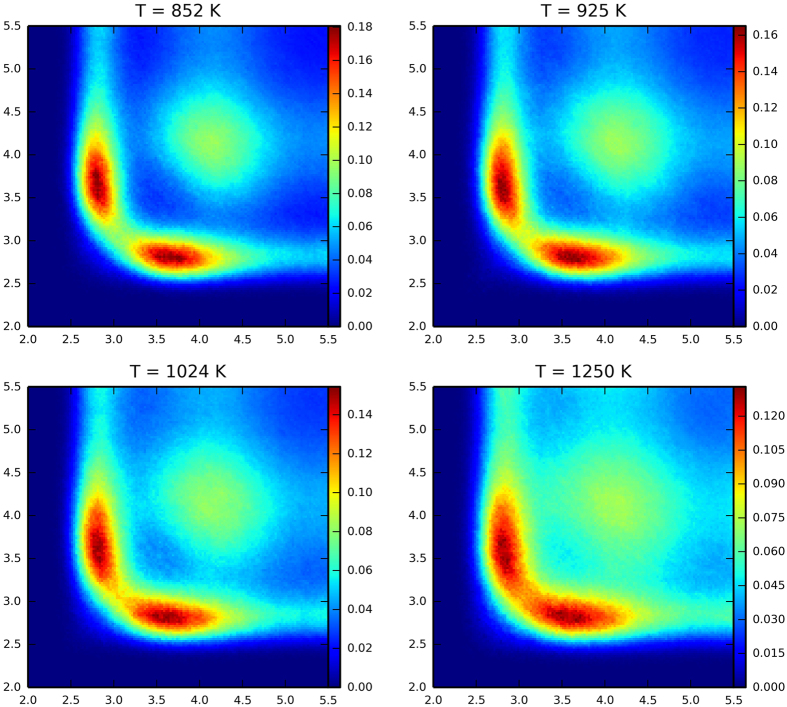
Total angular-limited three-body correlation (ALTBC) of Ge_2_Sb_2_Te_5_ at temperatures *T* = 852 K, 925 K, 1024 K, and 1250 K, obtained from the vdW-DF2 AIMD simulations. At the lowest temperature considered, two well-defined peaks indicative of Peierls distortion are observed. At higher temperature, the distortion becomes less pronounced. In particular, at *T* = 1250 K, the two peaks start to merge.

We also compute the bond angle distributions (BADs) (shown in Fig. 4) and the angular-limited three-body correlations (ALTBCs) from the AIMD trajectories (see Fig. 5). We consider both total BADs and partial BADs resolved for different central atoms. All of the total distribution functions display a peak centered at 90°, which is indicative of predominant (defective) octahedral coordination. The height of the peak decreases with increasing temperature. At the same time, the probability of observing bond angles below 80° and above 110° becomes more significant. The partial BADs for central Te are similar to the total ones, whereas the Sb BADs display a narrower main peak. On the other hand, the main peak of the Ge BADs is shifted to larger angles, especially at lower temperatures. This shift is due to the presence of a fraction of Ge atoms with tetrahedral coordination, characterized by bond angles around 104°. These structures contain at least one Ge-Ge or Ge-Sb bond. In fact, the BADs resolved over different atomic sequences shown in the supplement (Figs S4–7) clearly indicate that all the sequences containing Ge-Ge or Ge-Sb are peaked at angles around 105°–110°.

**Figure 6:**
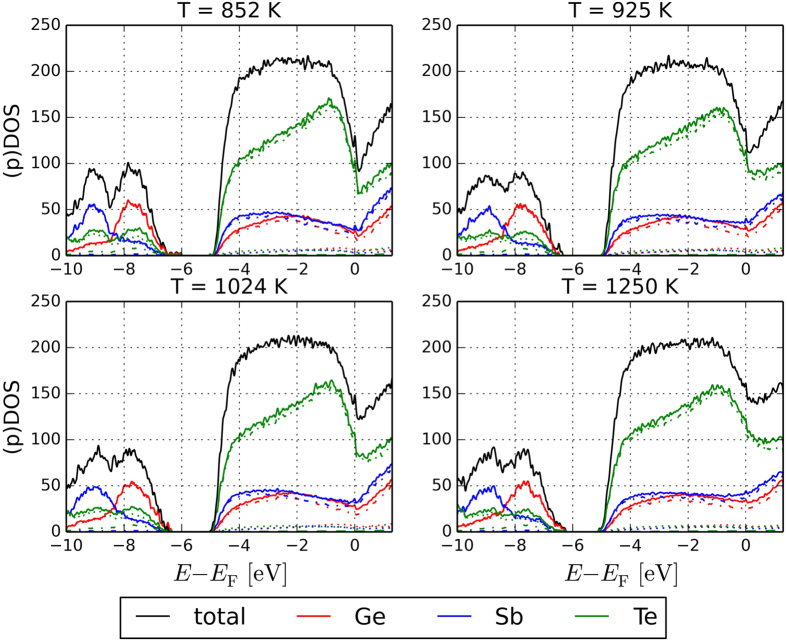
Electronic density of states (DOS) of liquid Ge_2_Sb_2_Te_5_ at different temperatures extracted from the vdW-DF2 AIMD simulations. The DOS projected (pDOS) onto the (*s*), (*p*) and (*d*) orbitals (dotted, dashed-dotted and dashed lines respectively) of the 3 atomic species is also included. The plots show that the DOS near the Fermi energy *E*_*F*_ has predominant (*p*) character, whereas the (*s*) orbitals mostly contribute to the states below −6 eV (notice that, in this region, the total DOS of Ge and Sb atoms basically coincides with the pDOS onto the (*s*) states).

The ALTBC expresses the probability of having a bond of a given length *r*_1_ almost aligned with a bond of length *r*_2_. We include angular deviations smaller than 25° in the calculation of the probability. The ALTBC distributions exhibit two peaks corresponding to alternating short and long bonds, indicative of Peierls distortion. Interestingly, the distortion appears to decrease upon increasing temperature. More specifically, the height of the two peaks decreases, as well as the distance between them. Partial ALTBC plots are shown in Figs S8–12. Similar effects have been previously observed in liquid GeTe and have been ascribed to the progressive transition from a semiconducting to a metallic liquid^43^. To further elucidate this phenomenon, we calculate the electronic density of states (averaged over 10 time steps) at different temperatures. This quantity indeed shows a pseudogap at the Fermi energy at low temperatures, whereas, at higher temperatures, the dip tends to disappear (see Fig. 6).

Finally, we compute the primitive ring statistics, which provides information about the intermediate range order. The relevant histograms are shown in Figs S13 and S14. We have calculated the number of rings both for fixed cutoff (3.2 Å) and by employing the cutoffs determined from the partial distribution functions included in Table 2. The most common ring structure is the 5-membered ring, as already discussed in ref. 28 for the supercooled liquid state. This is particularly evident if a fixed cutoff is used. In amorphous GST, 4-membered rings are instead predominant^14^,^15^,^16^. However, 4-membered rings become more numerous at the highest temperature considered (1250 K). Large rings up to 12-membered rings are abundant as well. Overall, an increase in the number of short and long rings is observed with increasing temperature, but only if variable cutoff lengths are used. We also consider ABAB rings, where A stands for Ge or Sb atoms and B denotes Te atoms. In amorphous GST, these rings constitute the majority of 4-membered rings. In our models of the liquid state, the fraction of 4-membered ABAB rings oscillates around 15–25% (using variable cutoffs) and 10–15% (for fixed cutoff).

### Kinetic properties

**Figure 7:**
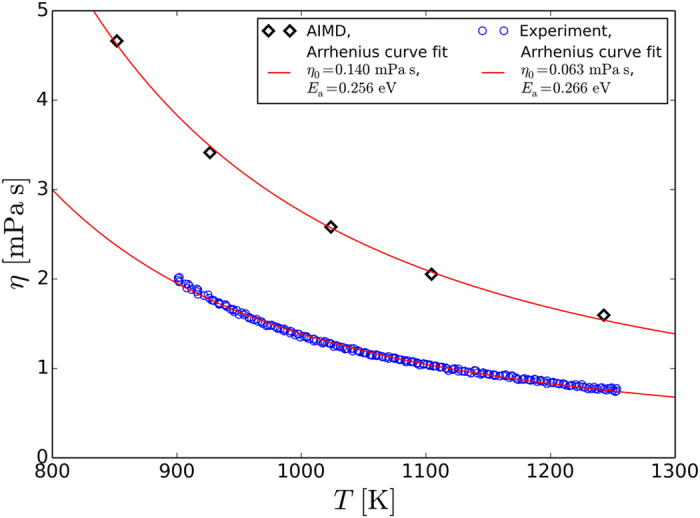
Dynamic viscosity *η* of liquid Ge_2_Sb_2_Te_5_ measured experimentally (blue circles) and calculated from the diffusivity derived from the vdW-DF2 AIMD trajectories by applying Stokes-Einstein relation (black diamonds). The lines are the fits with the Arrhenius equation 
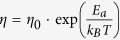
. For the theoretical viscosity, one obtains *η*_0_ = 0.140 mPa s, *E*_a_ = 0.256 eV; fitting of the experimental data instead yields *η*_0_ = 0.063 mPa s and *E*_a_ = 0.266 eV.

The diffusion coefficients are computed by applying Einstein’s formula,


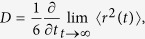


where 〈*r*^2^(*t*)〉 is the mean-squared distance over which the atoms have moved in the time interval *t*. The latter quantity is calculated by averaging over time and particles. The viscosity *η* is obtained from the diffusion coefficients using the Stokes-Einstein relation,


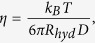


where *R*_*hyd*_ is the hydrodynamic radius. We estimate *R*_*hyd*_ from the simulations of GeTe carried out in ref. 31. We expect that *R*_*hyd*_ for Ge_2_Sb_2_Te_5_ should not differ significantly from the GeTe value. Notice that the Stokes-Einstein relation may break down at low temperatures close to the glass transition temperature due to the high fragility of the supercooled liquid state of GeTe and Ge_2_Sb_2_Te_5_^29^,^31^. Nonetheless, the relation is definitely valid in the weakly supercooled regime and above the melting temperature. In the temperature interval considered, the viscosities range from 1.6 mPa s to 4.8 mPa s. These values are larger than the experimental viscosities we have measured by the method of oscillating cup, as shown in Fig. 7 (see also Table S1 for the experimental data and Figs S15–19 for the mean-squared displacement plots). The latter quantities vary between 0.8 and 2.0 mPa s in the temperature range between 901 K and 1253 K. In the next section, we discuss possible origins of the mismatch. Nonetheless, we point out that the experimental and theoretical activation energies *E*_a_ are very similar. Fitting the two data sets with the Arrhenius equation 
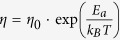
, where *η*_0_ is a constant representing the asymptotic viscosity at infinite temperature, *k*_*B*_ is the Boltzmann constant, and *T* is the absolute temperature, one obtains *η*_0_ = 0.140 mPa s, *E*_a_ = 0.256 eV (simulations) and *η*_0_ = 0.063 mPa s, *E*_a_ = 0.266 eV (experiments). The fit quality of *R*^2^ = 0.99205 for the experimental data (*R*^2^ = 0.98963 for fit of the AIMD results) shows that the Arrhenius law describes the temperature dependence of the dynamic viscosity for liquid Ge_2_Sb_2_Te_5_ very well.

## Discussion and Conclusions

In this work, we have determined the structural and dynamic properties of liquid Ge_2_Sb_2_Te_5_ in the temperature range between and 850 K and 1250 K. For this purpose, we have performed AIMD and RMC simulations, as well as neutron and X-ray diffraction, oscillating-cup viscometer and γ-ray attenuation experiments. In our AIMD simulations, we have mainly employed the vdW-DF2 functional^39^, which includes non-local correlations and, thus, provides a better description of van der Waals interactions than standard local and semi-local functionals. In ref. 41 vdW-DF2 simulations were shown to yield accurate models of amorphous GeTe. Our work indicates that this functional is also suitable to describe the liquid state of PCMs. Recently, semi-empirical van der Waals corrections^40^ in combination with gradient-corrected functionals have been employed to investigate liquid^27^ and amorphous^44^ GeTe_4_, as well as selected Ge-Sb-Te liquids with high Te content^28^. These corrections have also been shown to improve the level of agreement between calculations and experiments for said chalcogenides, although, for amorphous GeTe, their inclusion appears to be less effective^41^. Qualitatively speaking, both semi-empirical and *ab initio* van der Waals functionals lead to more sharply defined atomic connectivities and neighbour shells.

As far as the dynamical properties are concerned, there is qualitative, but not quantitative, agreement between simulations and experiments, in that the experimental viscosities are smaller than the theoretical ones in the temperature range considered. An important source of discrepancy probably lies in the approximations inherent in the exchange-correlation functional employed. In principle, even small deviations between the experimental and computational energy barriers for migration, *E*_a_, can lead to large deviations in the diffusion coefficients and viscosities. Our results indicate that the two activation energies differ by less than 5%. Interestingly, there are large differences in the prefactors *η*_0_: the theoretical estimate is more than twice as large as the experimental one. Nevertheless, we should point out that the theoretical *E*_a_ and *η*_0_ are affected by significant statistical uncertainties because of the small number of data points. It is not obvious what the effect of non-local functionals or Grimme corrections on the kinetic properties should be. In fact, for liquid GeTe_4_, the latter corrections yield smaller diffusion coefficients and, thus, larger viscosities than plain GGA simulations^27^. This point deserves further investigation. Another source of error is the finite size effects due to the periodic boundary conditions. These effects originate from the fact that a diffusing particle sets up a slowly decaying hydrodynamic flow, which, in a periodic system, brings about an interference between the particle and its periodic images^45^. Consequently, one obtains smaller diffusion coefficients as compared to the “real” values corresponding to the thermodynamic limit. Hence, larger simulation cells are expected to reduce the discrepancy with experiments. A third error source is caused by the use of thermostats. This technical point is discussed in the Methods section.

Importantly, the small (high-temperature) activation energy for viscosity we have obtained, *E*_a_ = 0.256 eV, is in agreement with previous results about similar phase-change materials^29^,^30^,^31^. Notice that, at lower temperatures, in the deep supercooled regime, there is a dramatic change in the activation energy. This behaviour is due to the high fragility^29^ of Ge_2_Sb_2_Te_5_ and is ultimately responsible for the remarkable stability of the amorphous state at room temperature a crucial property for applications of PCMs in memory devices.

## Methods

### *Ab-initio* molecular dynamics simulations

The AIMD constant-volume (NVT) simulations are carried out with the QUICKSTEP code included in the CP2K package^46^. We use the very efficient AIMD method developed by Kühne *et al.*^47^ This method is intrinsically dissipative and is employed in combination with Langevin thermostats, so that the simulations sample the canonical ensemble^47^,^48^. Notice that, in general, stochastic thermostats can affect the dynamical properties of the system significantly. CP2K uses a mixed Gaussian and plane-wave basis set. In our simulations, Kohn-Sham orbitals are expanded in a Gaussian-type basis set of triple-zeta plus polarization quality, whereas the charge density is expanded in plane waves, with a cutoff of 300 Ry. Scalar-relativistic Goedecker^49^ pseudopotentials are used. The Brillouin zone is sampled at the *Γ* point.

### Reverse Monte-Carlo simulations

Reverse Monte Carlo simulation is performed using rmc++ code^50^. The simulation box consists of 20250 atoms. Starting atomic configuration is a random distribution of atoms with the following minimum interatomic distance allowed in the model: 2.1 Å for Ge-Ge, 2.2 Å for Ge-Te and Ge-Sb, 2.4 Å for Sb-Sb, Te-Te, and Sb-Te. The atoms are moved randomly to optimize the fit to the experimental XRD and ND structure factors and to all of six partial pair distribution functions obtained by AIMD simulations.

### Samples preparation

The Ge_2_Sb_2_Te_5_ master alloys are prepared from proper quantities of pure elements (all 99.999%). An alloy for isotopic substitution neutron diffraction is prepared from ^70^Ge (enrichment 96.5%) and natural Sb and Te. Because of the high vapour pressure of Sb and Te, the alloys are synthesized in closed quartz ampoules. The ampoules are previously evacuated and filled with Ar to a pressure of about 250 mbar at room temperature. The alloys are melted and homogenized by isothermal heating at 1073 for 5 hours.

### X-ray diffraction experiment

X-ray diffraction experiment is carried out at the BW5 experimental station^51^ at the DORIS synchrotron storage ring, DESY, Hamburg, Germany. The sample is filled and sealed into a thin-walled quartz glass capillary (diameter −2 mm, wall thickness–about 0.02 mm). The sample is heated by a halogen spot-light heater. The energy of the X-ray radiation is 123.5 keV. The size of the incident beam is 1 × 4 mm^2^. Raw data is corrected for detector dead-time, background, polarization, absorption, variations in detector solid angle, and incoherent scattering. The corrected intensity is normalized to a Faber-Ziman^52^ total structure factor using X-ray atomic form factors given by Waasmaier and Kirfel^53^.

### Neutron diffraction with isotopic substitution

Neutron diffraction measurements are carried out at the Near and InterMediate Range Order Diffractometer^54^ (NIMROD) at the ISIS Second Target Station Facility of the Rutherford Appleton Laboratory, Oxford, UK. The samples are sealed under vacuum in quartz-glass tubes (4.95 mm outer diameter and 0.38 mm wall thickness) made for nuclear magnetic resonance spectroscopy (Hilgenberg GmbH, Malsfeld, Germany). The quartz-glass tube with the sample is inserted in a vanadium cylindrical cell (diameter of 6 mm; wall thickness of 40 μm), which is then placed in the middle of a vertical resistance-heating furnace. The sample temperature is kept constant with an accuracy of ±1 K during the isothermal measurements. The beam size is 8 × 30 mm^2^. Time-of-flight data are collected over a range of diffraction vector *q* between 0.1 and 30 Å^−1^. However, the useful range of *q* is limited to about 15 Å^−1^ because of the neutron absorption resonances in the sample occurring at high energies. ND measurements are also performed for the empty quartz-glass tube, the empty furnace, the empty spectrometer and the 6 mm thick vanadium rod for the purpose of instrument calibration and data normalization. Raw data are treated using the *Gudrun* program^55^ based upon algorithms in the ATLAS package^56^.

### Density measurements

The density of liquid Ge_2_Sb_2_Te_5_ alloy is determined by a high energy γ-ray attenuation method. About 110 Mbq of ^137^Cs is used as a γ-ray source of 662 keV and a NaI scintillation counter is used as a γ-ray detector. Ge_2_Sb_2_Te_5_ specimen is sealed in an ampoule made of fused quartz with a diameter of 1.1 cm and a length of 2.388 ± 0.002 cm. The length of the ampoule is determined by measuring the linear attenuation coefficient of Hg at room temperature. The linear attenuation coefficients for pure elements are measured using freshly powdered specimens compressed into a cylindrical steel tube. They are 0.007005 ± 0.000004 (m^2^/kg) for Ge, 0.007329 ± 0.000008 (m^2^/kg) for Sb and 0.007182 ± 0.000003 (m^2^/kg) for Te. The uncertainty of the experimental values associated with counting statistics is less than 0.11%. The total experimental uncertainty is estimated to be less than 0.5%^57^.

### Viscosity measurements

The dynamic viscosity of liquid Ge_2_Sb_2_Te_5_ alloy is measured using an oscillating-cup viscometer described elsewhere^58^. The sample is sealed in a quartz crucible with 22.5 mm inner diameter under Ar atmosphere as above. The ampoule is inserted into a graphite holder connected to a torsion wire. The oscillating unit is situated in a high-vacuum chamber with a Mo-wire resistance heater and appropriate shielding. The temperature of the sample is measured with uncertainty of about ±5 K by a thermocouple placed at the bottom of the sample holder. The damped torsion oscillations excited by a PC-controlled mechanical unit are detected by means of a laser-beam/photodiode unit. The system is heated at a rate of 5 K min^−1^ to 1253 K, held for 1 hour at the constant temperature for homogenization, and cooled at a rate of 1 K min^−1^. Ten to twelve oscillations are registered for each measured point during cooling of liquid Ge_2_Sb_2_Te_5_ alloy down to a solidification point at 900 K. The dynamic viscosity of the melt is determined from the measured oscillations using an equation given by Roscoe *et al.*^59^ and modified by Brooks *et al.*^60^. The relative uncertainty of the dynamic viscosity is less than 10%, as estimated by Gruner *et al.*^58^. The measurements repeated several times showed a very good reproducibility.

## Additional Information

**How to cite this article**: Schumacher, M. *et al.* Structural, electronic and kinetic properties of the phase-change material Ge_2_Sb_2_Te_5_ in the liquid state. *Sci. Rep.* **6**, 27434; doi: 10.1038/srep27434 (2016).

## Supplementary Material

Supplementary Information

## Figures and Tables

**Table 1:** Experimental values of the molar volume and number density of liquid Ge_2_Sb_2_Te_5_ as a function of temperature.

*T* [K]	*V* [cm^3^/mol]	*ρ* [nm^−3^]	*T* [K]	*V* [cm^3^/mol]	*ρ* [nm^−3^]
893	19.582	3.075	1113	19.942	3.020
913	19.607	3.071	1133	19.977	3.014
933	19.632	3.067	1153	20.016	3.009
953	19.660	3.063	1173	20.057	3.002
973	19.683	3.060	1193	20.106	2.995
993	19.714	3.055	1213	20.154	2.988
1013	19.751	3.049	1233	20.190	2.983
1033	19.784	3.044	1253	20.242	2.975
1053	19.815	3.039	1273	20.292	2.968
1073	19.851	3.034	1293	20.337	2.961
1093	19.893	3.027			

The experimental uncertainty is less than 0.5%^57^.

**Table 2:** Total (*N*_tot_) and partial (*N*_Ge,_ *N*_Sb_, *N*_Te_) coordination numbers in liquid Ge_2_Sb_2_Te_5_ at different temperatures, extracted from the vdW-DF2 AIMD trajectories.

Temperature	Species	*N*_tot_	*N*_Ge_	*N*_Sb_	*N*_Te_
*T* = 852 K	Ge	4.33	0.31 (3.16)	0.45 (3.26)	3.57 (3.50)
	Sb	4.13	0.45 (3.26)	0.59 (3.48)	3.09 (3.50)
	Te	3.49	1.43 (3.50)	1.23 (3.50)	0.83 (3.50)
*T* = 925 K	Ge	4.32	0.36 (3.05)	0.37 (3.24)	3.59 (3.50)
	Sb	4.17	0.37 (3.24)	0.86 (3.50)	2.94 (3.50)
	Te	3.57	1.43 (3.50)	1.18 (3.50)	0.96 (3.50)
*T* = 1024 K	Ge	4.36	0.33 (3.05)	0.48 (3.30)	3.55 (3.50)
	Sb	4.23	0.48 (3.30)	0.82 (3.50)	2.93 (3.50)
	Te	3.66	1.42 (3.50)	1.17 (3.50)	1.06 (3.50)
*T* = 1250 K	Ge	4.50	0.44 (3.13)	0.67 (3.35)	3.38 (3.50)
	Sb	4.33	0.67 (3.35)	0.84 (3.50)	2.82 (3.50)
	Te	3.78	1.35 (3.50)	1.13 (3.50)	1.30 (3.50)

The coordination numbers are calculated by integrating the partial *g*_*ij*_(*r*) shown in Figs 2 and S1–3, up to cutoffs corresponding to their first minimum. If *g*_*ij*_(*r*) does not display any minimum up to 3.5 Å (e.g. *g*_TeTe_(*r*)), the cutoff is set to this value. The cutoff values are listed in brackets.
