# Supplementary material for: Structural, electronic and kinetic properties of the phase-change material Ge2Sb2Te5 in the liquid state
Source: Sci Rep. 2016 Jun 8;6:27434. doi: 10.1038/srep27434 (PMC4897623; doi:10.1038/srep27434)
Supplement: Supplementary Information [file srep27434-s1.pdf]

Structural, electronic and kinetic properties of the phase-change material $Ge_2Sb_2Te_5$ in the liquid state

Mathias Schumacher[1], Hans Weber[2,3], Pál Jóvári[4], Yoshimi Tsuchiya[5], Tristan G. A. Youngs[6], Ivan Kaban[2*] and Riccardo Mazzarello[1,7]*

[1] Institute for Theoretical Solid State Physics, RWTH Aachen University, 52056 Aachen, Germany

[2] IFW Dresden, Institute for Complex Materials, PO Box 270116, 01171 Dresden, Germany

[3] TU Dresden, Institut für Strukturphysik, 01062 Dresden, Germany

[4] Wigner Research Centre for Physics, Institute for Solid State Physics and Optics, PO Box 49, 1525 Budapest, Hungary

[5] Department of Physics, Faculty of Science, Niigata University, Ikarashi 2-8050, Niigata 950-21, Japan

[6] ISIS Facility, STFC Rutherford Appleton Laboratory, Harwell Oxford, Didcot, UK

[7] JARA-FIT and JARA-HPC, RWTH Aachen University, 52056 Aachen, Germany

* Corresponding authors. Email addresses: i.kaban@ifw-dresden.de, mazzarello@physik.rwth-aachen.de

In this supplement, we show the calculated partial pair distribution functions of liquid $Ge_2Sb_2Te_5$ (Figures S1-3), partial bond angle distributions (Figures S4-7), statistics of the number of primitive rings (Figures S8 and S9), and the total and partial angular-limited three-body correlation (ALTBC) plots of $Ge_2Sb_2Te_5$,obtained from the molecular dynamics trajectories at different temperatures (Figures S10-14). In addition, we show plots of the mean square displacements (S15-19), from which we extracted the viscosity as a function of temperature, as given in Figure 7. We also include a table (Table S1) with the experimental viscosities measured by the method of oscillating cup (also plotted in Figure 7).

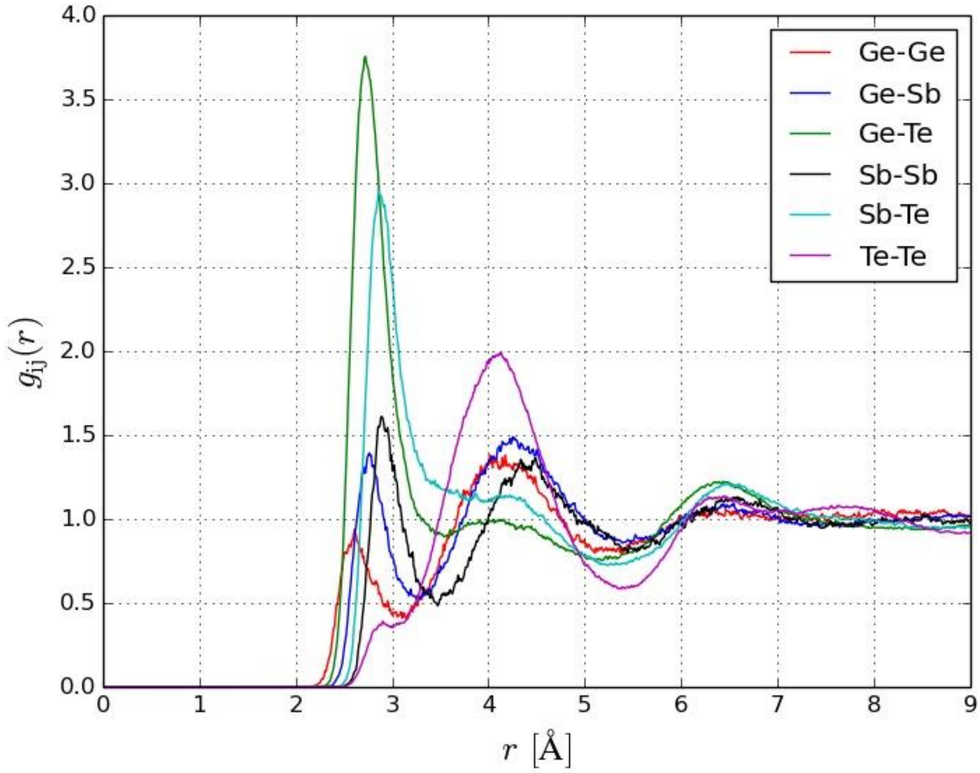

Figure S1: Partial pair distribution functions, $g_{ij}(r)$, of liquid $Ge_2Sb_2Te_5$ at T = 852 K, obtained by AIMD simulation.

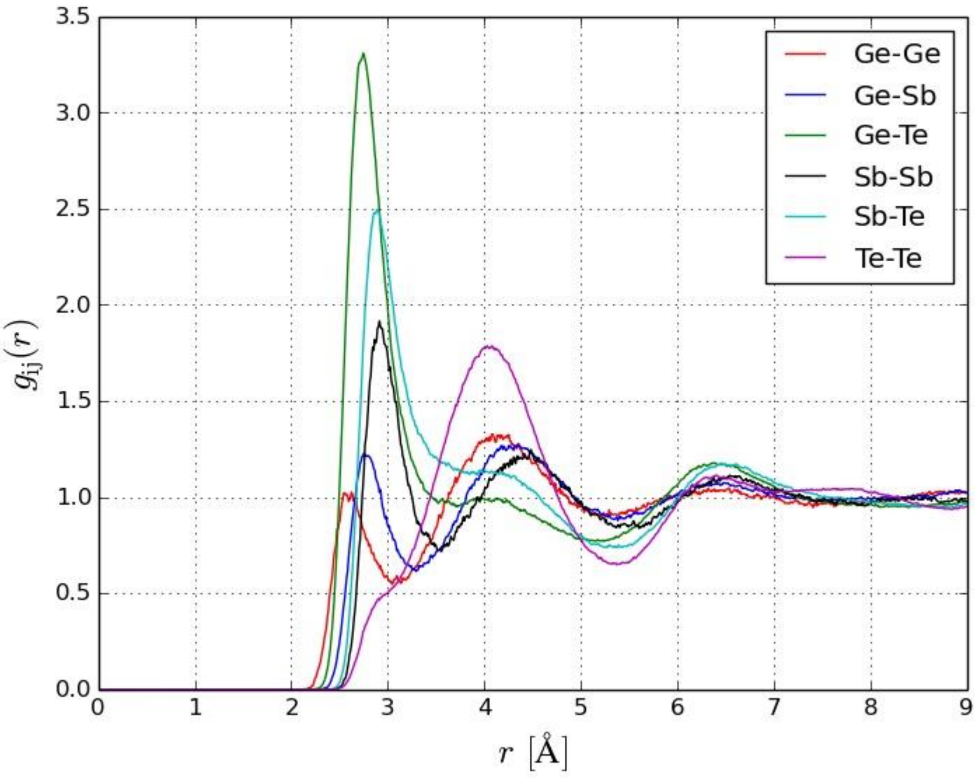

Figure S2: Partial pair distribution functions, $g_{ij}(r)$, of liquid $Ge_2Sb_2Te_5$ at T = 1024 K, obtained by AIMD simulation.

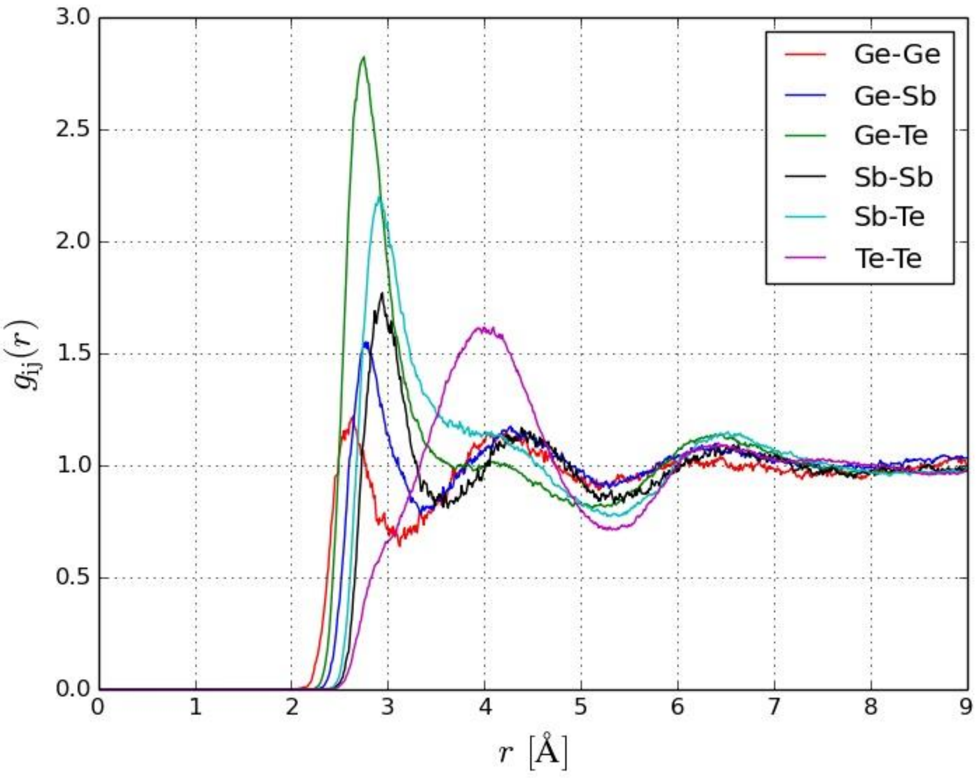

Figure S3: Partial pair distribution functions, $g_{ij}(r)$, of liquid $Ge_2Sb_2Te_5$ at T = 1250 K, obtained by AIMD simulation.

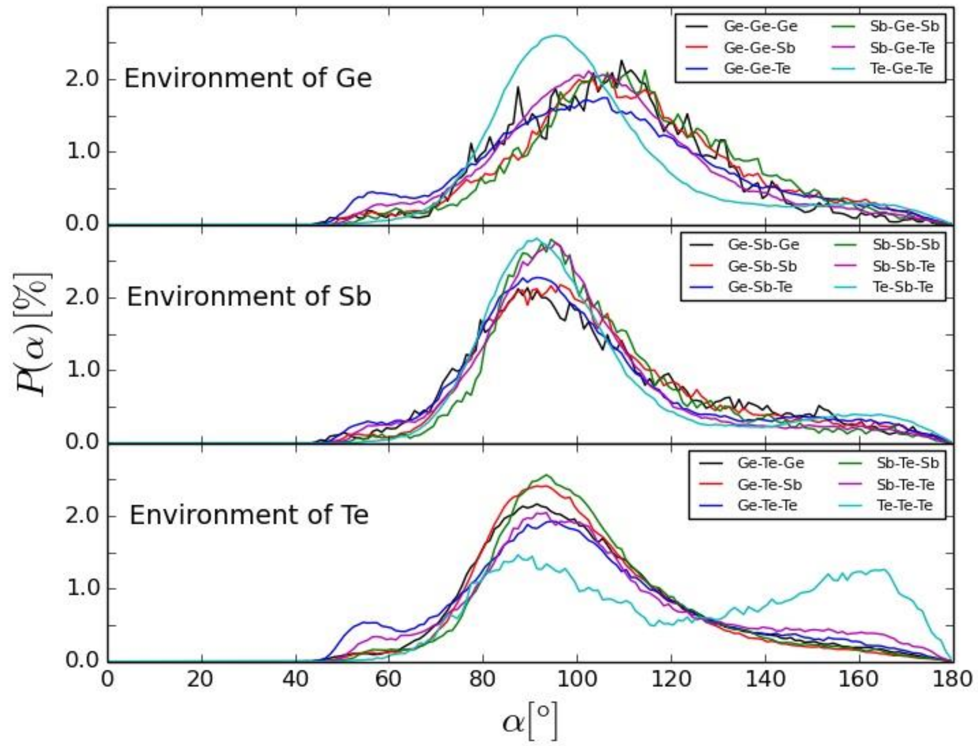

Figure S4: Bond angle distributions, *P(α)*, for different central atoms resolved over different bond configurations at temperature *T* = 852 K.

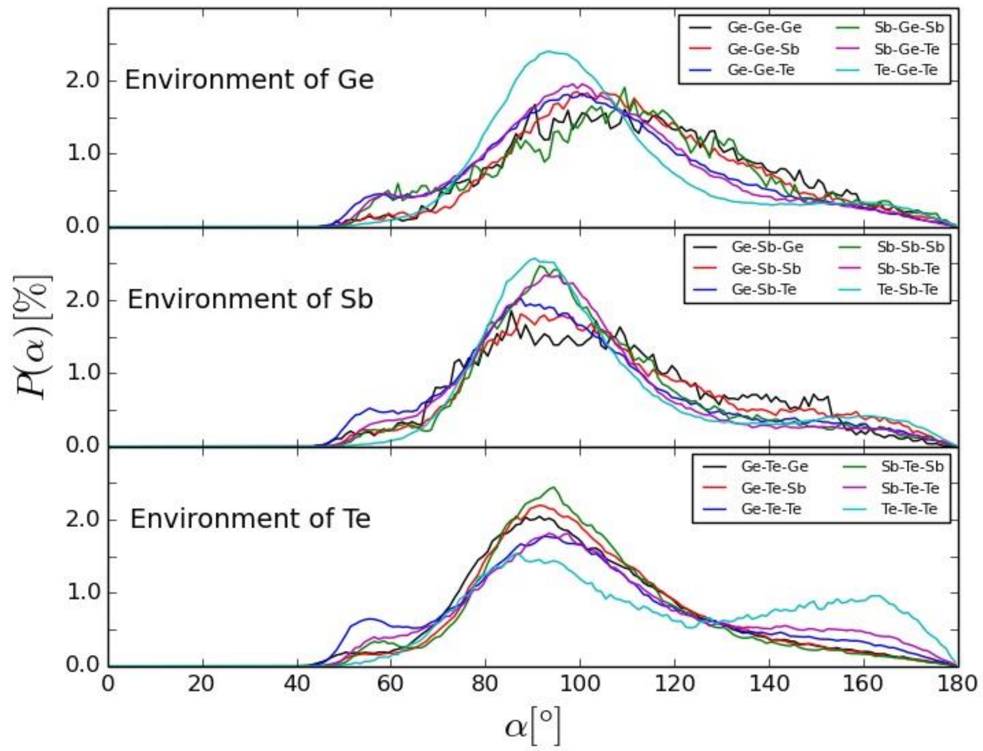

Figure S5: Bond angle distribution, *P(α)*, resolved for different central atoms resolved over different bond configurations, at temperature *T* = 925 K.

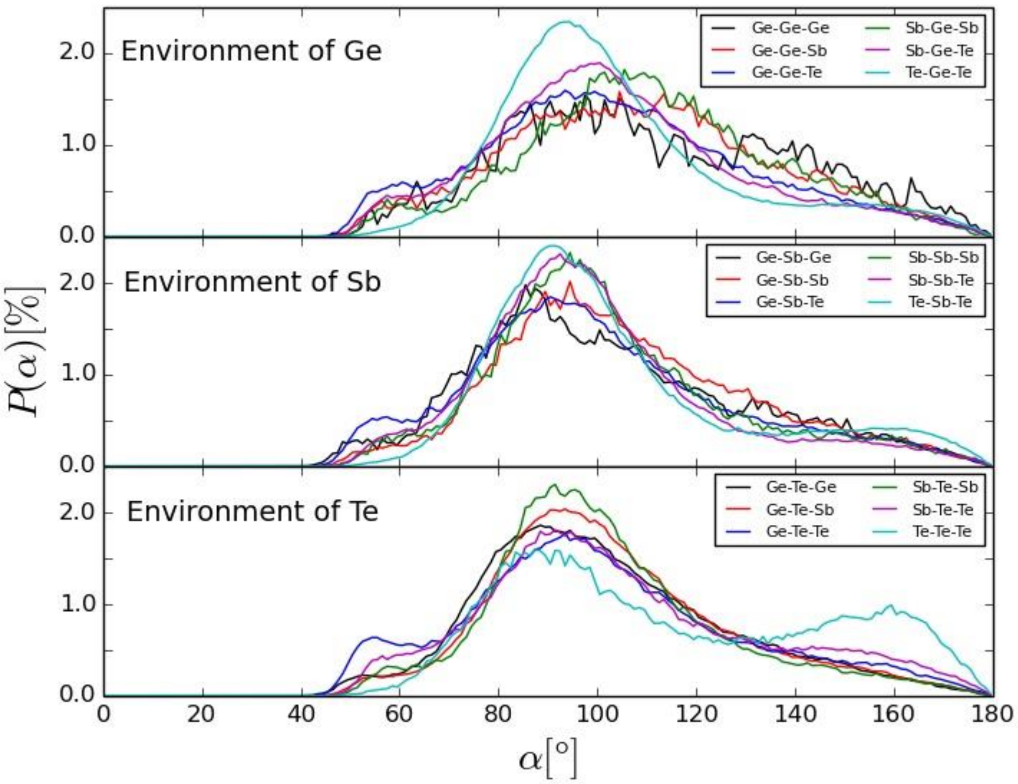

Figure S6: Bond angle distribution, *P(α)*, for different central atoms resolved over different bond configurations, at temperature *T* = 1024 K.

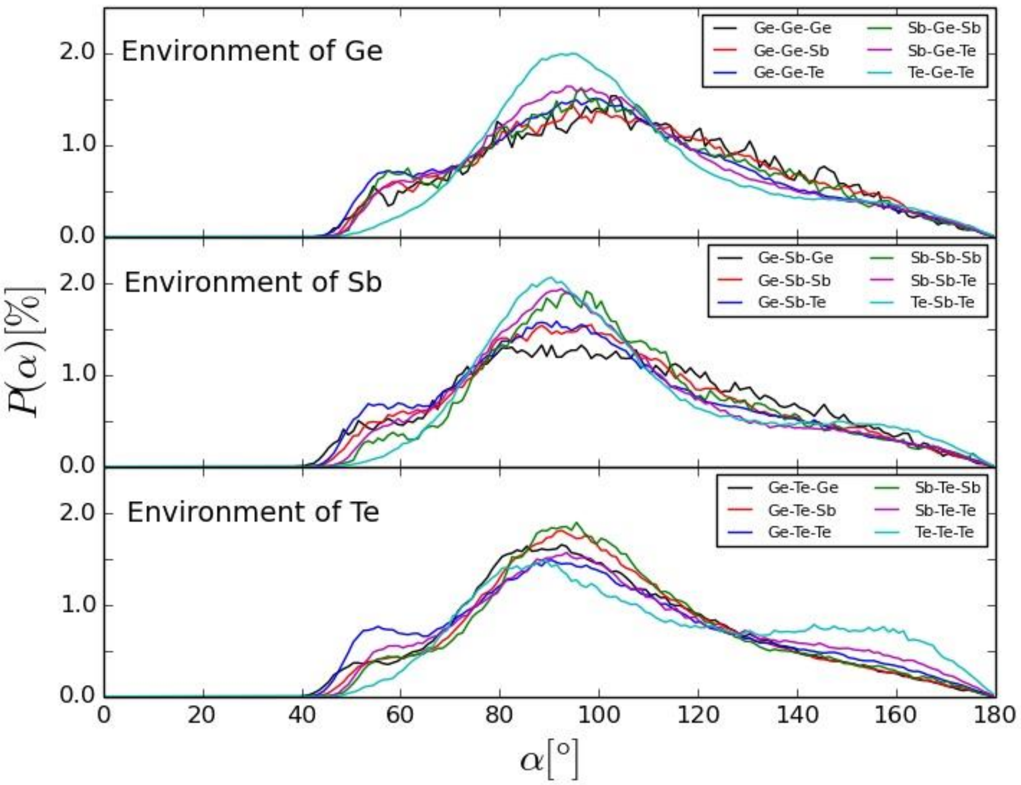

Figure S7: Bond angle distribution, *P(α)*, for different central atoms resolved over different bond configurations, at temperature *T* = 1250 K.

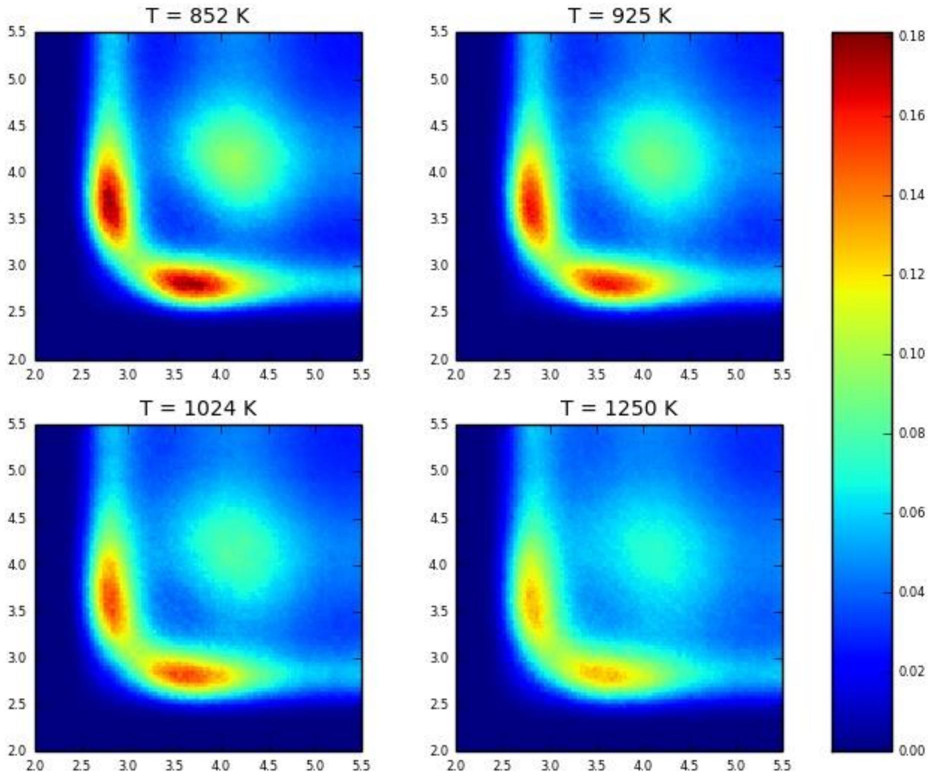

Figure S8: Total angular-limited three-body correlation (ALTBC) of $Ge_2Sb_2Te_5$ at temperatures T = 852 K, 925 K, 1024 K, and 1250 K. At the lowest temperature considered, two well defined peaks indicative of Peierls distortion are observed. At higher temperature the distortion becomes less pronounced. In particular, at T = 1250 K the two peaks start to merge together. This figure shows the same data as Fig. 4 in the main text but with a common color scale for the panels.

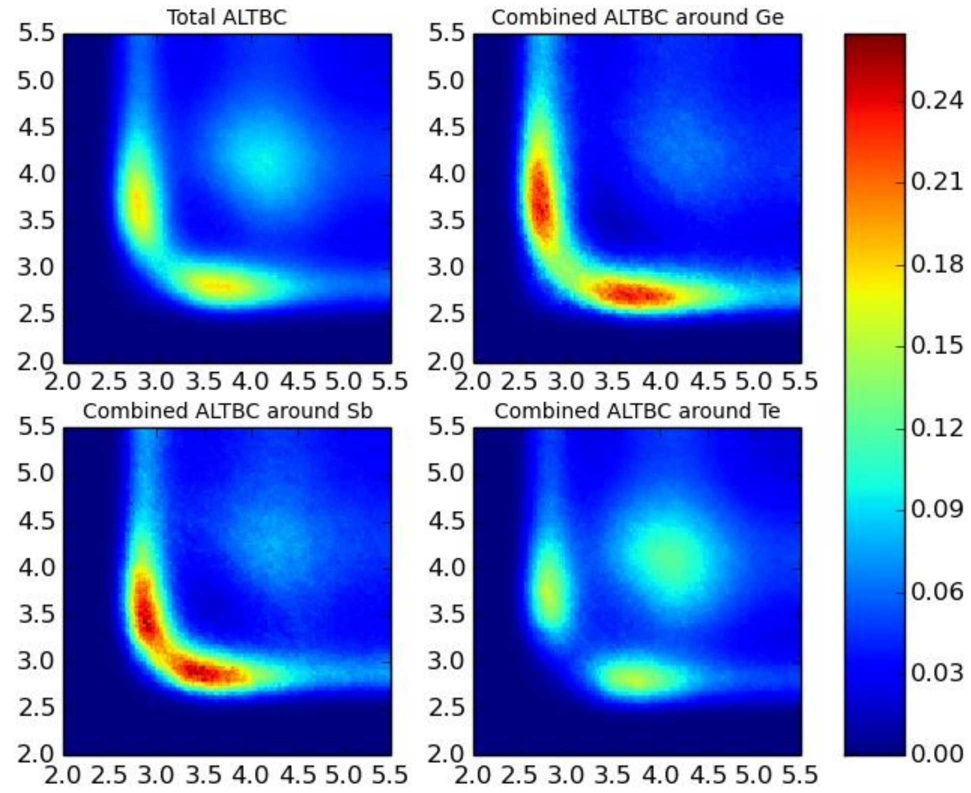

Figure S9-a: Total and partial ALTBC plots of $Ge_2Sb_2Te_5$ at T = 852 K, common color scale.

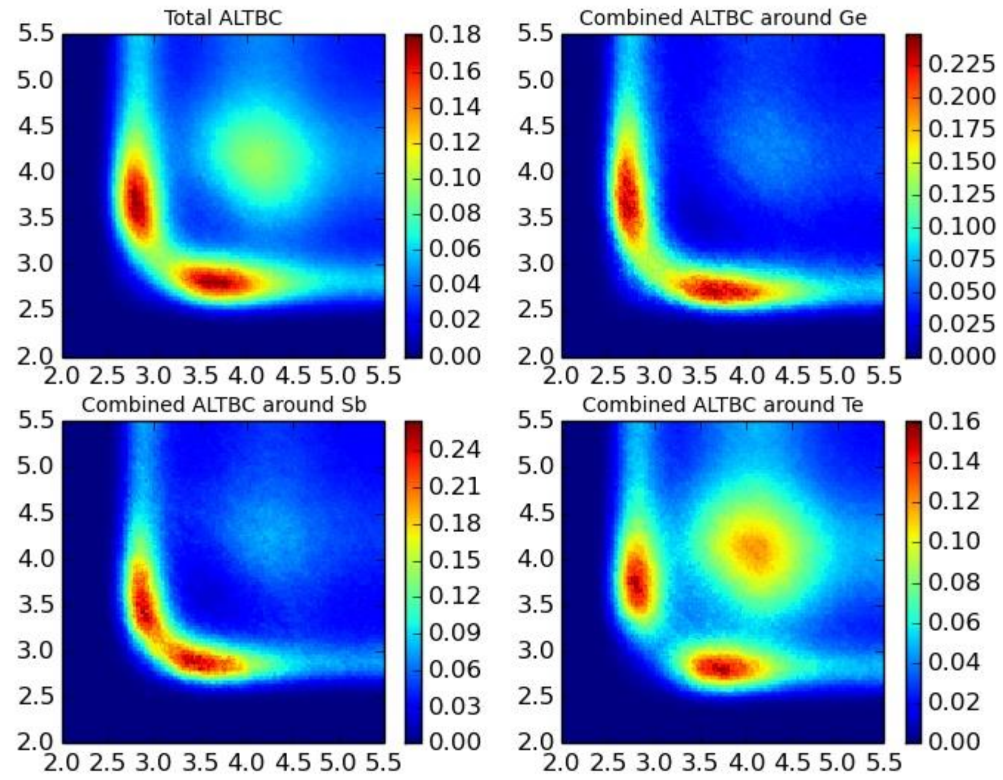

Figure S9-b: Total and partial ALTBC plots of $Ge_2Sb_2Te_5$ at T = 852 K, separate color scales.

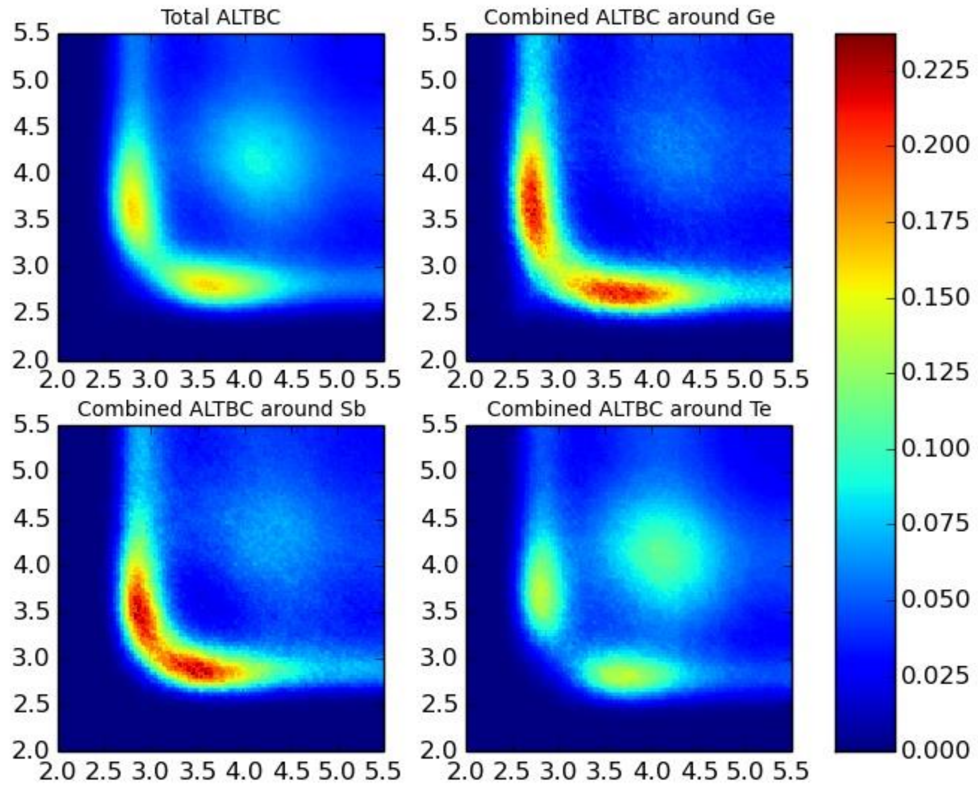

Figure S10-a: Total and partial ALTBC plots of $Ge_2Sb_2Te_5$ at T = 925 K, common color scale (normalized to the maximum value of the ALTBC distribution at this temperature).

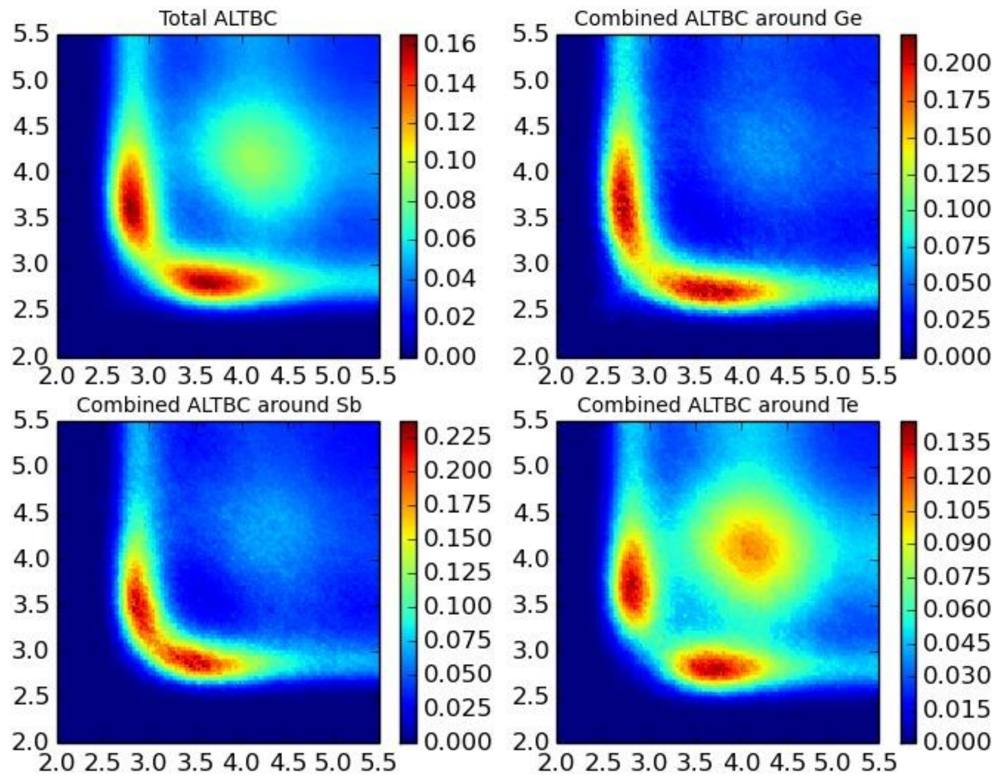

Figure S10-b: Total and partial ALTBC plots of $Ge_2Sb_2Te_5$ at T = 925 K, separate color scales.

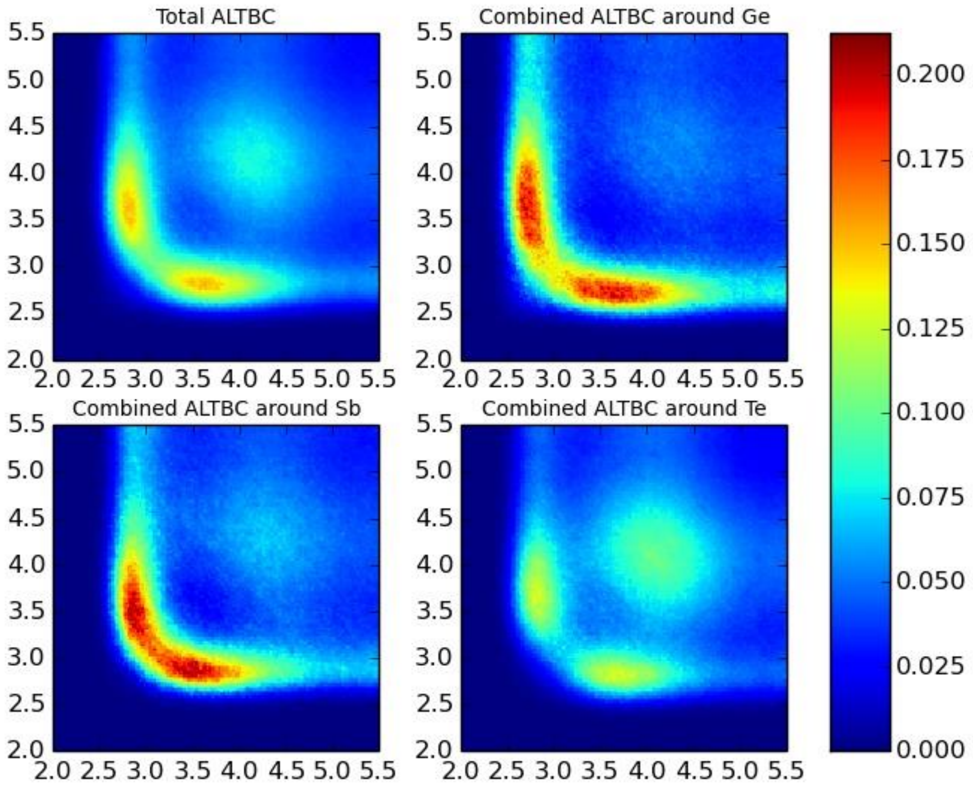

Figure S11-a: Total and partial ALTBC plots of $Ge_2Sb_2Te_5$ at T = 1024 K, common color scale (normalized to the maximum value of the ALTBC distribution at this temperature).

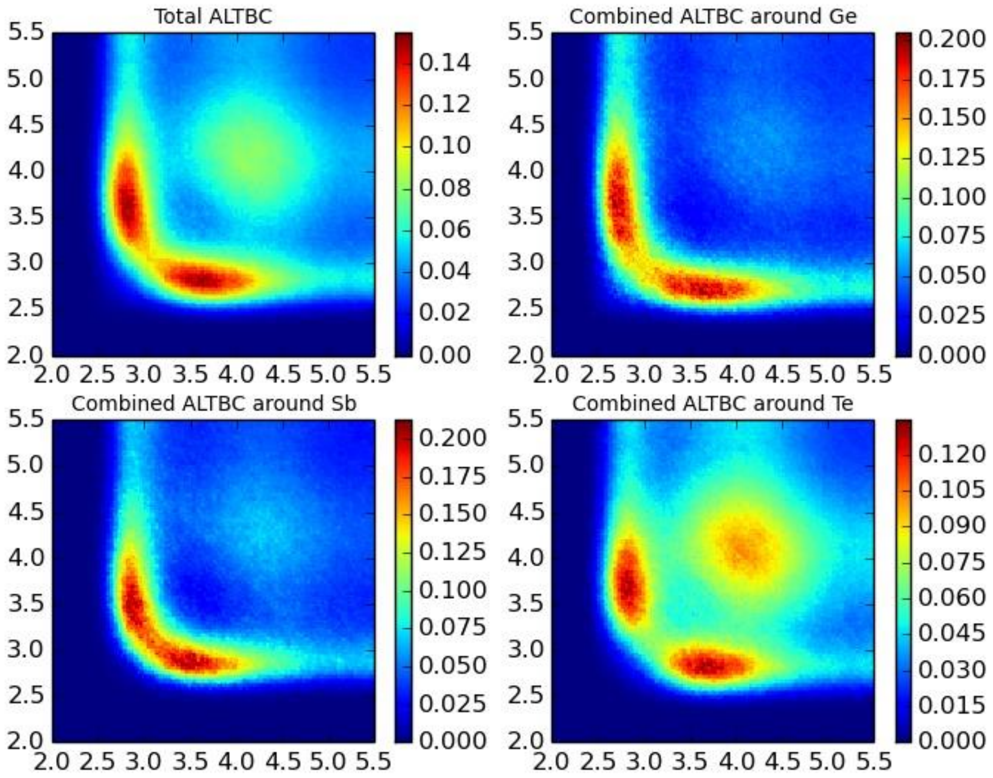

Figure S11-b: Total and partial ALTBC plots of $Ge_2Sb_2Te_5$ at T = 1024 K, separate color scales.

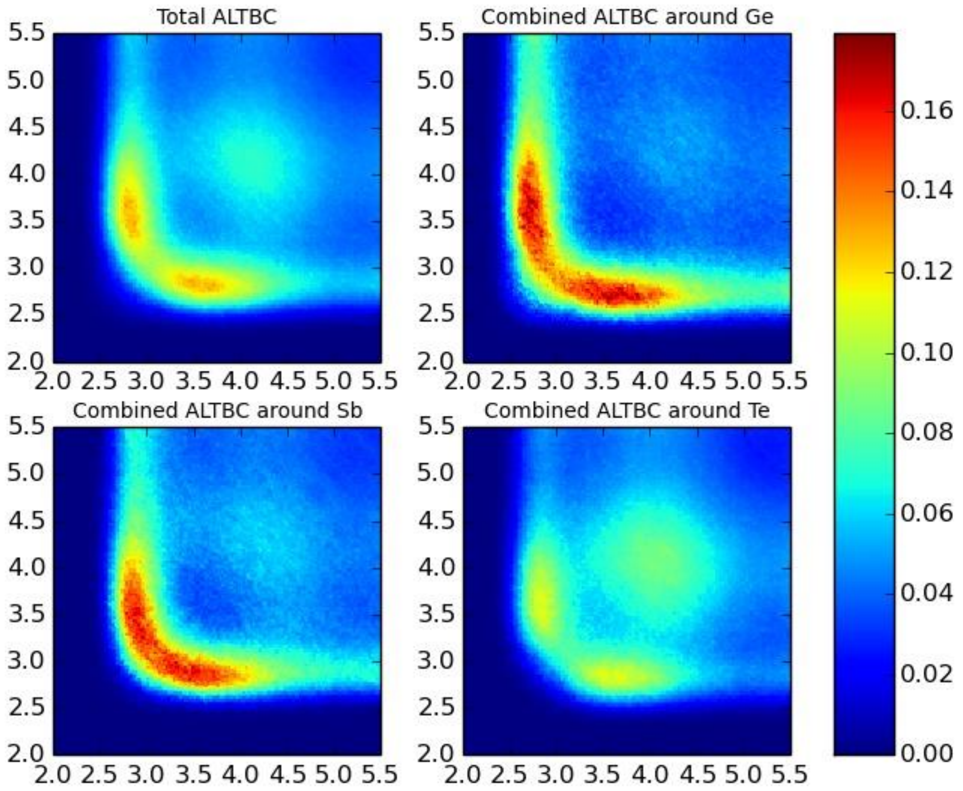

Figure S12-a: Total and partial ALTBC plots of $Ge_2Sb_2Te_5$ at T = 1250 K, common color scale (normalized to the maximum value of the ALTBC distribution at this temperature).

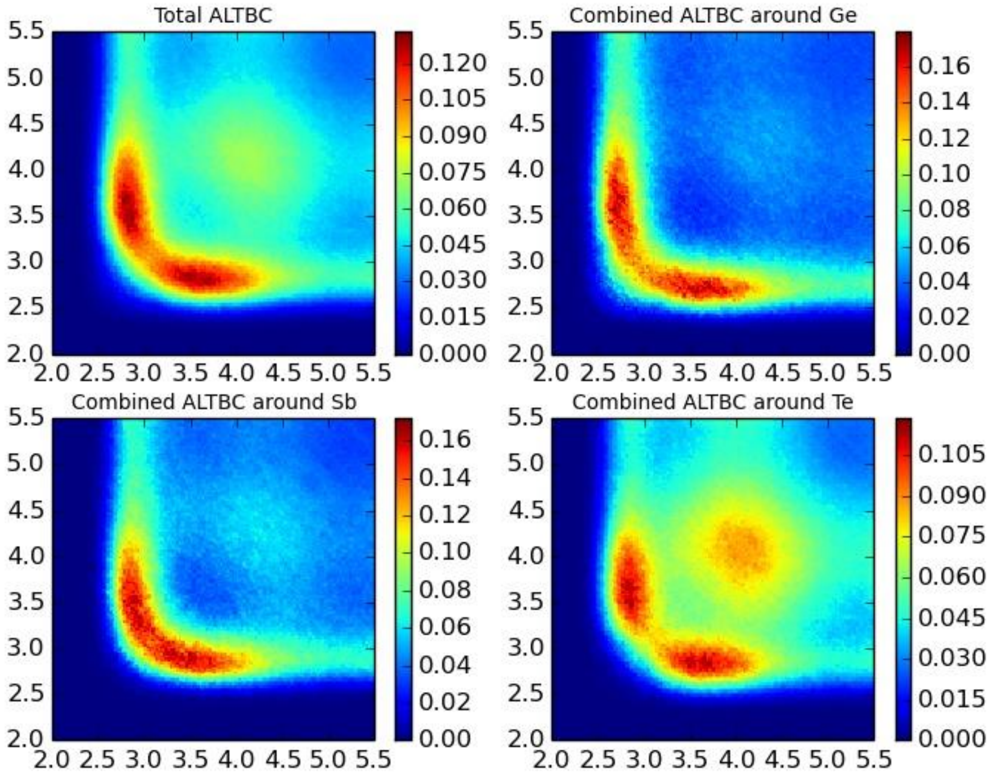

Figure S12-b: Total and partial ALTBC plots of $Ge_2Sb_2Te_5$ at T = 1250 K, separate color scales.

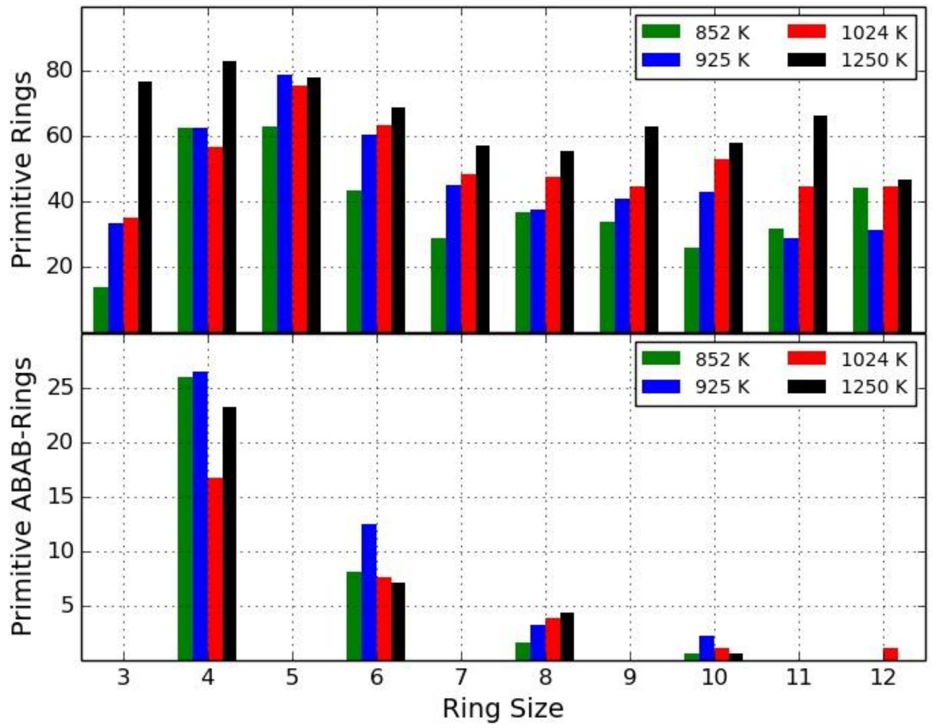

Figure S13: Numbers of primitive rings in liquid $Ge_2Sb_2Te_5$ at temperatures ranging from 852 K to 1250 K. The upper plot shows the total number of primitive rings, whereas the lower one is restricted to rings with alternating sequence of atomic species (ABAB-rings, where A denotes Ge or Sb and B stands for Te). The positions of the first minima of the partial distribution functions (see Figure 2 in the main text and Figs. S1-S3) were chosen as cutoffs for the inter-particle distances. The cutoffs are shown in Table 2 of the main text.

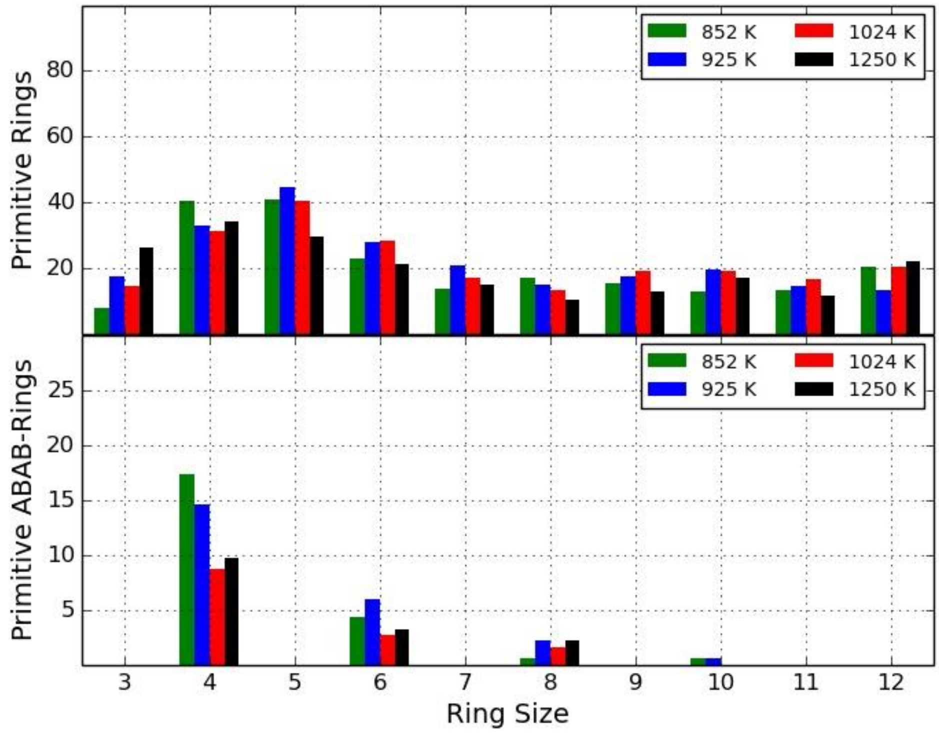

Figure S14: Statistics of primitive rings obtained from the same MD trajectories considered in Fig. S4. In contrast to the analysis of Fig. S4, a constant cutoff of 3.2 Å for the particle-particle distance has been used.

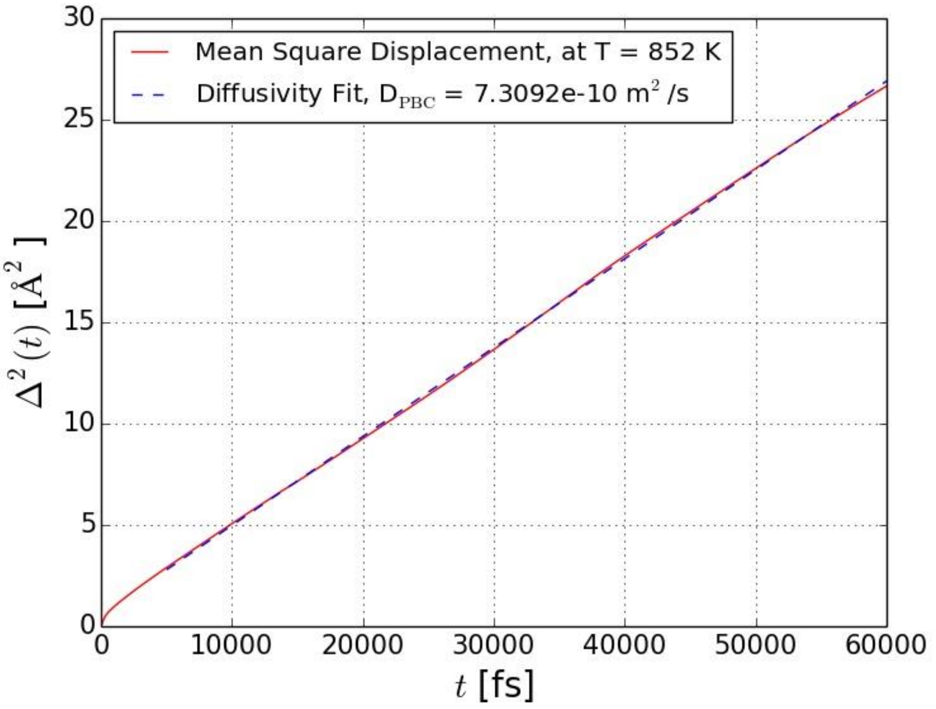

Figure S15: Mean square displacement (MSD) obtained from the MD simulation at T = 852 K. The diffusivity inside the cubic supercell with periodic boundary conditions, D_{PBC}, is determined from a linear fit (blue dashed line) of the MSD data (red line).

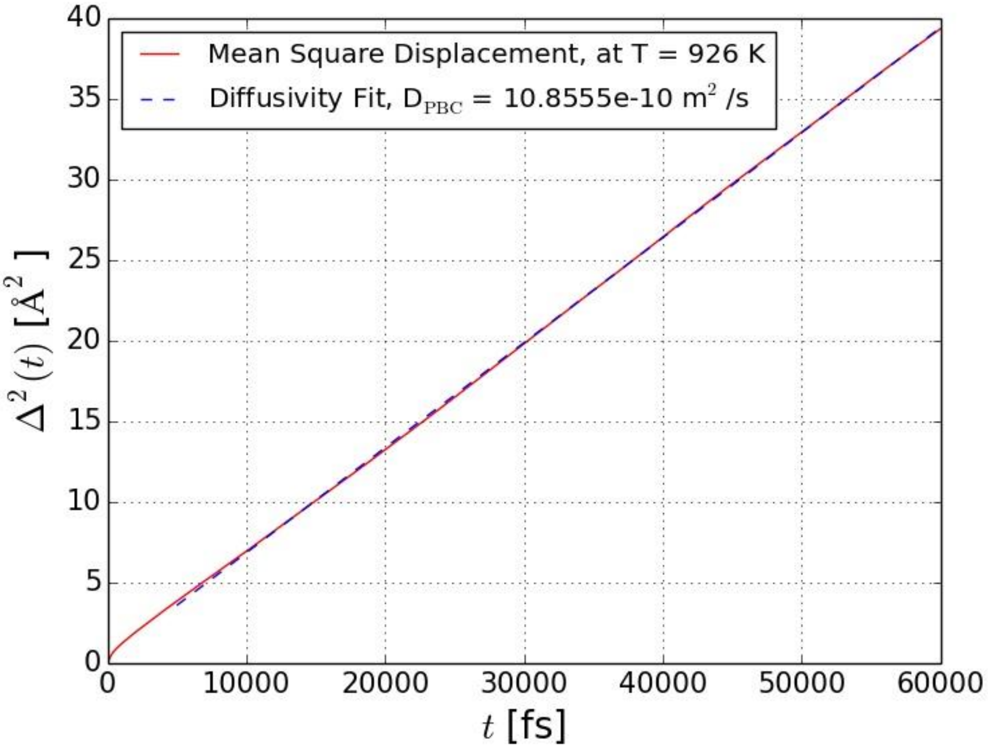

Figure S16: MSDs obtained from MD simulations at T = 926 K. The diffusivity D_{PBC} is determined from a linear fit (blue dashed line) to the MSD data (red line).

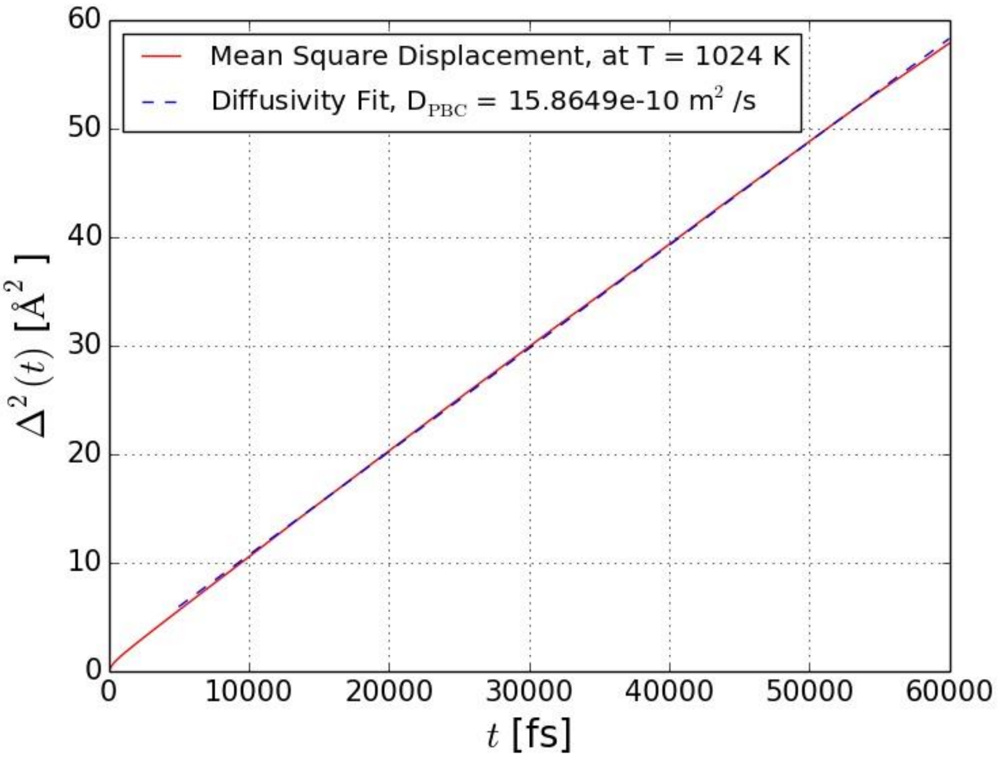

Figure S17: MSDs obtained from MD simulations at T = 1024 K. The diffusivity D_{PBC} is determined from a linear fit (blue dashed line) of the MSD data (red line).

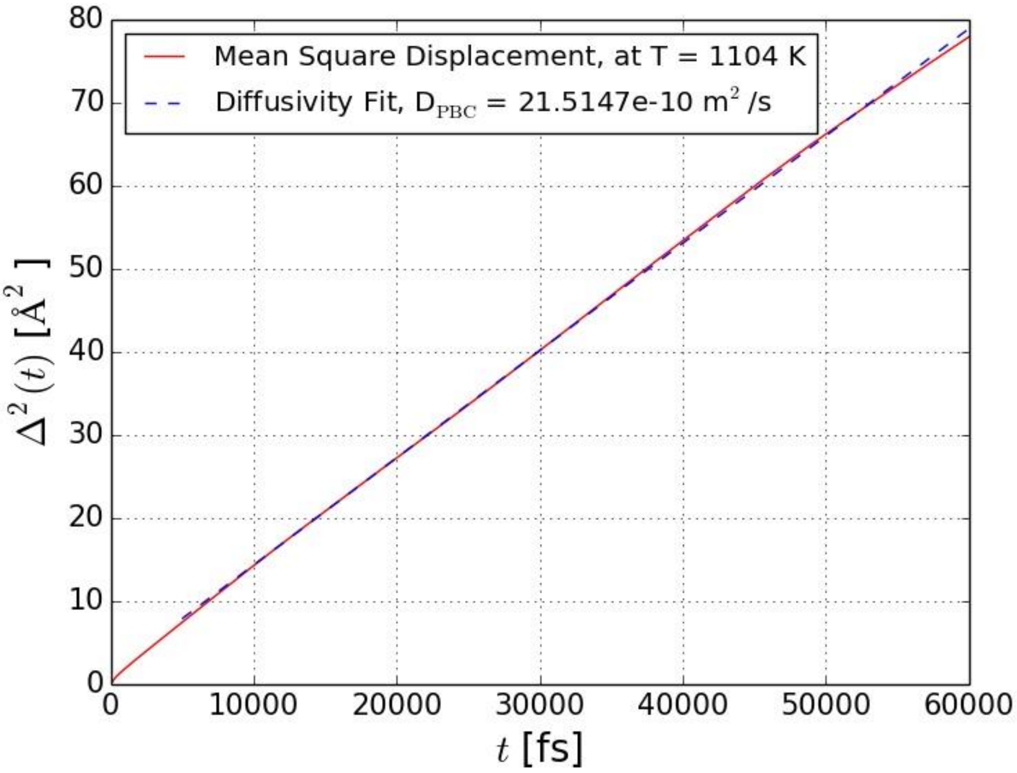

Figure S18: MSDs obtained from MD simulations at T = 1104 K. The diffusivity D_{PBC} is determined from a linear fit (blue dashed line) of the MSD data (red line).

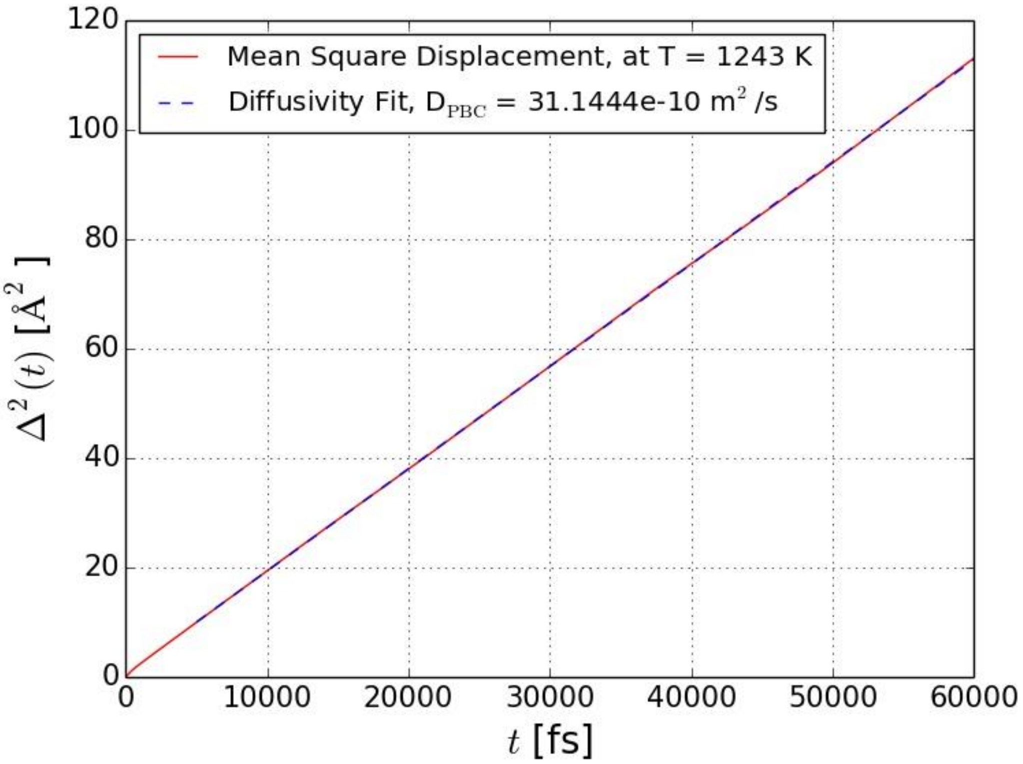

Figure S19: MSDs obtained from MD simulations at T = 1243 K. The diffusivity D_{PBC} is determined from a linear fit (blue dashed line) of the MSD data (red line).

T [K]	η [mPa s]	T [K]	η [mPa s]	T [K]	η [mPa s]	T [K]	η [mPa s]	T [K]	η [mPa s]	T [K]	η [mPa s]
901	2.010	960	1.555	1022	1.283	1083	1.089	1144	0.934	1205	0.836
901	1.984	962	1.543	1023	1.282	1084	1.072	1145	0.945	1207	0.830
902	2.019	964	1.550	1024	1.274	1085	1.088	1146	0.952	1207	0.826
902	1.968	965	1.527	1026	1.246	1088	1.073	1149	0.941	1210	0.830
907	1.945	968	1.522	1028	1.258	1089	1.078	1150	0.933	1211	0.826
908	1.899	968	1.519	1029	1.247	1090	1.074	1151	0.932	1212	0.814
909	1.942	969	1.502	1031	1.241	1093	1.052	1154	0.932	1215	0.805
910	1.925	972	1.477	1033	1.237	1093	1.064	1155	0.919	1216	0.810
912	1.882	973	1.486	1035	1.216	1095	1.037	1156	0.922	1217	0.814
916	1.889	975	1.481	1036	1.223	1098	1.045	1159	0.914	1220	0.819
916	1.861	976	1.486	1037	1.222	1098	1.055	1159	0.915	1221	0.797
917	1.878	979	1.468	1040	1.22	1101	1.046	1162	0.924	1223	0.801
917	1.830	981	1.446	1041	1.195	1103	1.031	1164	0.929	1225	0.821
922	1.814	981	1.460	1042	1.222	1103	1.040	1164	0.914	1226	0.794
922	1.815	983	1.445	1045	1.200	1106	1.024	1167	0.897	1228	0.786
925	1.824	986	1.442	1046	1.194	1107	1.029	1169	0.911	1230	0.771
926	1.787	986	1.419	1047	1.185	1108	1.029	1169	0.920	1231	0.790
928	1.764	987	1.429	1050	1.174	1111	1.013	1172	0.897	1233	0.773
929	1.764	991	1.395	1051	1.179	1112	1.016	1173	0.883	1235	0.792
930	1.757	991	1.417	1052	1.172	1113	1.015	1174	0.876	1236	0.770
934	1.735	992	1.412	1055	1.152	1116	1.007	1177	0.877	1238	0.765
934	1.724	995	1.375	1056	1.155	1117	0.997	1178	0.877	1240	0.780
934	1.716	997	1.386	1057	1.144	1118	0.977	1179	0.886	1241	0.786
939	1.704	998	1.383	1060	1.143	1121	0.986	1182	0.877	1243	0.766
939	1.713	999	1.350	1060	1.153	1122	0.995	1183	0.870	1245	0.757
940	1.692	1002	1.360	1062	1.146	1123	0.998	1184	0.866	1246	0.776
943	1.665	1004	1.358	1065	1.133	1126	0.996	1187	0.881	1248	0.778
945	1.662	1004	1.346	1065	1.129	1126	0.969	1188	0.874	1249	0.785
945	1.653	1006	1.334	1068	1.122	1129	0.969	1189	0.853	1250	0.756
946	1.633	1010	1.326	1070	1.110	1131	0.971	1192	0.867	1252	0.741
948	1.649	1010	1.330	1070	1.133	1131	0.966	1192	0.858	1253	0.750
950	1.604	1010	1.305	1073	1.120	1134	0.960	1195	0.852	1253	0.774
952	1.623	1012	1.311	1075	1.110	1136	0.950	1197	0.861	1253	0.776
954	1.598	1015	1.302	1075	1.120	1136	0.946	1197	0.831		
955	1.613	1017	1.282	1078	1.100	1139	0.978	1200	0.841		
958	1.565	1018	1.293	1079	1.096	1140	0.974	1202	0.846		
958	1.572	1019	1.303	1080	1.097	1141	0.949	1202	0.839		

Table S1: Experimental values of the dynamic viscosity $\eta(T)$ for liquid $Ge_2Sb_2Te_5$ measured with an oscillating-cup viscometer. The relative uncertainty of the dynamic viscosity is less than 10%. The uncertainty of the temperature is ±5K.
